# Projection-specific circuits of retrosplenial cortex with differential contributions to spatial cognition

**DOI:** 10.1038/s41380-024-02819-8

**Published:** 2024-11-07

**Authors:** Xiaoxiao Lin, Ali Ghafuri, Xiaojun Chen, Musab Kazmi, Douglas A. Nitz, Xiangmin Xu

**Affiliations:** 1 Department of Anatomy & Neurobiology, School of Medicine, University of California, Irvine, CA 92697 USA; 2 Department of Cognitive Science, University of California, La Jolla, San Diego, CA 92093 USA; 3 The Center for Neural Circuit Mapping, University of California, Irvine, Irvine, CA 92697 USA

**Keywords:** Neuroscience, Biotechnology

## Abstract

Retrosplenial cortex (RSC) is a brain region involved in neuropsychiatric and neurodegenerative disorders. It has reciprocal connections with a diverse set of cortical and subcortical brain regions, but the afferent structure and behavioral function of circuits defined by its projection-specific sub-populations have yet to be determined. The corticocortical connections between RSC and secondary motor cortex (M2), as well as corticothalamic connections between RSC and anterodorsal thalamus (AD) have been hypothesized to function as semi-independent, but parallel pathways that impact spatial information processing in distinct ways. We used retrograde and anterograde viral tracers and monosynaptic retrograde rabies virus to quantitatively characterize and compare the afferent and efferent distributions of retrosplenial neuron sub-populations projecting to M2 and AD. AD-projecting and M2-projecting RSC neurons overlap in their collateral projections to other brain regions, but not in their projections to M2 and AD, respectively. Compared with AD-projecting RSC neurons, M2-projecting RSC neurons received much greater afferent input from the dorsal subiculum, AD, lateral dorsal and lateral posterior thalamus, and somatosensory cortex. AD-projecting RSC neurons received greater input from the anterior cingulate cortex and medial septum. We performed chemogenetic inhibition of M2- and AD-projecting RSC neurons and examined its impact on object-location memory, object-recognition, open-field exploration, and place-action association. Our findings indicate that inhibition of M2-projecting RSC neurons impairs object location memory as well as place-action association, while the RSC to AD pathway impacts only object-location memory. The findings indicate that RSC is composed of semi-independent circuits distinguishable by their afferent/efferent distributions and differing in the cognitive functions to which they contribute.

## Introduction

The retrosplenial cortex (RSC) is involved in diverse cognitive functions in primates and rodents, including spatial navigation, orientation, mnemonic processing, and planning [1–5]. Structural and functional alterations within the retrosplenial cortex have been linked to neurodegenerative and neuropsychiatric disorders [6–8]. In rodents, it is considered as a component of the posterior cingulate cortex, given its close proximity to the cingulate cortex, parietal cortex, and secondary visual cortex [9, 10]. The RSC contains two subregions: the granular area (RSCg) in the ventral region, corresponding to human Brodmann areas (BA) 29, and the dysgranular area (RSCd) in more dorsal region corresponding to BA 30 [11, 12]. The two areas are defined by the cytoarchitecture of pyramidal neurons in different layers [10] and their unique circuit connectivity patterns [13–15].

The RSC is known as a “hub” because of its extensive and diverse reciprocal connections with multiple cortical and subcortical brain regions, including the hippocampus and subicular complex [15–21]. However, the form and degree of integration of the multiple afferents and their relationships to RSC efferent sub-populations is not well understood. At one extreme, neuron sub-groups within RSCd and RSCg might obtain an even distribution of afferent inputs from all sources forming a complete integration of all inputs for all projection-specific sub-groups of RSC neurons. At another extreme, RSC neuron sub-groups defined by their efferent projections might obtain non-overlapping sets of afferents from among the different afferent sources. At the latter extreme, RSC would function as a set of distinct circuits each with responsiveness to different afferent sources of information. Some evidence for this model of RSC connectivity comes from a recent optogenetic study demonstrating the existence of parallel inputs from subiculum/thalamus and claustrum/cingulate cortex to different subpopulations of RSCg neurons [22].

Major RSC inputs are obtained from thalamus [23–25], cingulate cortex, orbitofrontal cortex, parietal cortex, visual cortex, septal nucleus, claustrum, and subregions of the subicular complex [12, 17]. Additionally, the RSC projects back to all these regions as well as the secondary motor cortex (M2) and entorhinal cortex. Afferent connectivity strength varies across its granular and dysgranular sub-regions. Specifically, the granular area expresses stronger connections with anterodorsal (AD) and anteroventral (AV) subregions of thalamus while the dysgranular area is more heavily connected with anteromedial (AM) and lateral dorsal nuclei (LD) [23, 26].

Recent studies have functionally dissected the cell-type-specific excitatory synaptic connectivity that constitutes the RSC to M2 circuit in mice [27]. RSC is associated with motor action selection based on recognition of familiar environmental contexts [28]. Many RSC neurons exhibit conjunctive tuning to left and right turning actions and the environmental locations where they occur [29]. Together, these studies suggest that the reciprocal connection between RSC and M2 might serve as a cellular mechanism for: 1) transforming integrated spatial information from hippocampus and subiculum and head orientation information from AD into navigational actions; and 2) associative learning of place-action contingencies.

Our objective in this work was to determine whether distinct projection-specific sub-populations of RSC can be considered as semi-independent circuits based on their afferent/efferent connectivity patterns and based on their contributions to behavior, memory, and spatial cognition. To investigate whether RSC connectivity is organized in a circuit-specific manner, we utilized genetically modified retrograde viral tracers, including a new variant of adenovirus-associated virus (rAAV2-retro-Cre) and rabies virus, as well as an anterograde synaptic terminal marker targeting AAV synaptoTAG2 [30–32]. Synaptic inputs to M2- and AD-projecting neurons differed significantly in connection strengths, with M2-projecting RSC neurons receiving much greater input from the dorsal subiculum, thalamus, and sensory cortex, compared to AD-projecting RSC neurons. In turn, AD-projecting RSC neurons receive much greater input from local RSC neurons and medial septum. While both M2- and AD-projecting RSC neurons show overlapping collateral connections to output regions other than M2 and AD, they are only sparsely colocalized within RSC. Thus, both the afferent and efferent compositions of M2- and AD-projecting RSC neurons evidence semi-independent circuit structure and function. To further understand the functional roles of these distinct circuits, we performed chemogenetic manipulations and behavioral tests, including object location memory (OLM), novel object recognition, anxiety-related tests, and a place-action navigational task. Consistent with a function for M2-projecting RSC neurons in transforming spatial cognition into action, our results indicate that inhibition of M2-projecting RSC neurons impairs object-location memory and action-location memory, whereas AD-projection neurons only affect object-location memory. Overall, these findings provide a strong foundation to consider the largely unexplored functional roles of these pathway-specific circuit connections in RSC.

## Material and methods

### Animals

Ethics approval and consent to participate: All experiments were conducted according to the National Institutes of Health guidelines for animal care and use and were approved by the Institutional Animal Care and Use Committee (IACUC, protocol # AUP-22-163) and the Institutional Biosafety Committee (IBC, protocol # BUA-R100) of the University of California, Irvine. Since this work did not involve human subjects, informed consent from participants was not applicable.

In the viral circuit tracing experiments, 2- to 4-month-old male C57BL/6 J mice were used to study RSC circuit connections and relevant behavioral studies. A total of 25 mice were used for the experimental procedures, including 15 for rabies tracing and 10 for AAV-tracing cases. To ensure the clarity and specificity of our analysis, only the 10 rabies tracing mice were included in the final CSI quantification. We selected only those starter neurons located in the RSC granular layer for our analysis, excluding cases that may have exhibited leakage into surrounding regions. Another cohort comprising male C57BL/6 J mice aged between 2- to 4-month-old, totaling 20 individuals was used to examine the functional roles of M2- and AD-projecting RSC neurons in the behavioral tests. See the text in S1 Table for detailed information.

### Viral injections

The protocol for viral injection was carried out by anesthetizing mice with 2–3% isoflurane for 5 minutes using an isoflurane tabletop unit with a 0.8 L/min oxygen flow rate. Subsequently, the mice were transferred to a rodent stereotaxic frame where they were anesthetized with a continuous flow of 1–1.5% isoflurane. After making a small incision in the head to resect the skin, the skull was exposed to reveal the landmarks of bregma and lambda. A digital Atlas-guided 3-axis micromanipulator was used to determine the coordinates of the injection site relative to the bregma and lambda. The virus was delivered into the brain by a picospritzer pressure injection. For pressure injection, a small drill hole was made in the skull above the injection site, exposing the pia surface. A glass pipette was then loaded with the virus and lowered into the brain at the appropriate coordinates. A picospritzer was used to pulse the virus into the brain at a rate of 20 to 30 nl/min with a 10-ms pulse duration. On the other hand, for iontophoresis, the virus was delivered with a positive 3-μA current in a cycle of 7 seconds “on” and 7 seconds “off” for 10 minutes. After the injection was completed, the injection pipette was left in the brain for 5 minutes to prevent backflow of the virus. Once the injection pipette was removed, the mouse was taken out of the stereotaxic frame and the incision was closed with tissue adhesive (3 M Vetbond, St. Paul, Minnesota, USA). Finally, mice were given an injection of Carprofen and returned to their home cages to recover.

#### rAAV2-retro virus

To test the colocalization of M2- and AD- projecting RSC neurons, we used fluorescent protein-expressing rAAV2-retro in the viral tracing experiments [30]. rAAV2-retro-mRuby (with a titer of 9 × 10^12^ genomic units per ml, custom packaged by the Center for Neural Circuit Mapping Viral Core, UC Irvine) or rAAV2-retro-EGFP (with a titer of 1.36 × 10^13^ genomic units per ml; Vigene, Plasmid# 105553) was injected into M2 (coordinates: AP: -0.22 mm; ML: -0.83 mm; DV: -1.18 mm) or AD (coordinates: AP: -0.82 mm; ML: -0.79 mm; DV: -2.91 mm) in C57BL/6 mice. The mice were then allowed to recover for three weeks before being perfused for tissue processing. To target projection-specific RSC subpopulations, we injected 0.1 μl of colorless rAAV2-retro-Cre (with a titer of 4.5 × 10^12^ genomic units per ml; Addgene; plasmid# 105553) into the M2 and AD regions of each C57 mouse. The C57 mice were then injected with different types of tracing viruses, depending on the specific tracing goals of each experiment.

#### Helper AAV and rabies viruses

The rabies virus (EnvA-RVΔG-DsRed) was produced locally at the University of California, Irvine’s Center for Neural Circuit Mapping using necessary cell lines and seeding viruses from E. Callaway’s group at the Salk Institute for Biological Studies. The injection of rAAV2-retro-Cre was done in the M2 or AD brain region prior to administrating the helper AAV8-hSyn-DIO-TC66T-2A-eGFP-2A-OG (0.1 μl, 9.5 × 10^12^ genome units per ml, SALK institute custom) into RSC via pressure injection on the same day. The coordinates of RSC relative to the bregma were AP: -2.46 mm, ML: −0.2 mm, DV: −0.93 mm (S1 Table). Following a three-week period after the helper AAV injection, which allowed the infected neurons to express high levels of RG and EGFP, we injected the pseudotyped G-deleted rabies virus (EnvA-SADΔG-RV-DsRed, 0.4 μl, approximately 2 × 10^7^ infectious units per ml) into the same target region as the helper AAV. The rabies virus was then given 9 days to replicate and retrogradely spread from targeted Cre+ cell types to directly connected presynaptic cells before the mice were perfused for tissue processing.

#### AAVDJ-hsyn-DIO-SynaptoTAG2-tdTomato

To examine the collateral projections of M2- and AD- projecting RSC neurons, we used a Cre-dependent anterograde-directed synaptoTAG2 virus (0.1 μl, 3 ×10^13^ infectious units per ml, from Wei Xu Lab in UT Southwest) and C57BL/6 mice. The injection of rAAV2-retro-Cre was done in the M2 or AD brain region prior to administrating the synaptoTAG2 virus (0.1 μl) in RSC. The injection coordinates for M2, AD and RSC are the same as those used in other experiments. AAV-DIO-synaptoTAG2 virus was allowed to express and spread to postsynaptic neurons for 3 weeks before the animals were perfused for tissue processing.

#### AAV2-DIO-hM4D-mCherry

To genetically inhibit the RSC neurons projecting to M2 or AD, wild-type C57BL/6 J mice were injected with rAAV2-retro-Cre bilaterally at ML: ±0.83 mm; AP: -0.22 mm; DV: -1.18 mm for M2 and ML: ±0.79 mm; AP: -0.82 mm; DV: -2.91 mm for AD. Following this, a delivery of 0.3 μl of AAV2-DIO-hM4D-mCherry (3.7 × 10^12^ genomic copies/ml; Addgene: 44362) was administered bilaterally in the same mouse to RSC at ML: ±0.2 mm; AP: −2.46 mm; DV: −0.93 mm. The mice were allowed to recover in their home cages for 3 weeks before behavior experiments.

### Histology and immunochemical staining

The mice were perfused with 5 ml of PBS and then with 25 ml 4% paraformaldehyde in PBS. The perfused brains were post-fixed in 4% paraformaldehyde and transferred to 30% sucrose in 1 X PBS after 24 hours. The frozen brains were sectioned coronally with a thickness of 30-μm using a microtome (Leica SM2010R, Germany). Every third section was mounted for examination of virally labeled neurons in various brain regions for computer-based analyses and neurochemical characterization of the labeled cells. To determine the neurochemical cell types of rAAV2-retro-Cre labeled postsynaptic neurons in M2 or AD brain regions, immunostaining for Cre was performed. A primary rabbit anti-Cre antibody (NOVUS, 1:500 dilution) was used, followed by a Cy5-conjugated donkey anti-rabbit secondary antibody (Jackson ImmunoResearch, West Grove, PA, USA, 1:200 dilution).

### Behavioral experiments

#### Drug

CNO (Enzo Life Sciences, Farmingdale, New York, USA; BML-NS105-0025) was dissolved in saline to make a concentration of 1 mg per ml on an experimental day. All the animals were administered with either CNO or saline intraperitoneally (i.p.) at 5 mg per kg 30 minutes before anxiety-like tests, the training session of the memory tests and daily test of the cross-maze test.

#### Behavioral tasks

Before experiments, mice were handled for seven days and habituated to intraperitoneal injections. Each animal then underwent a 30-minute habituation period in the testing room. Subsequently, a CNO injection was administered, and the mice were allowed an additional 30 minutes for acclimatization before beginning the experiments. Anxiety-like and memory tests followed previously established protocols, with minor modifications. Prior to the cross-maze test, all animals were water deprived, and their weight loss was monitored and controlled within 15% of their original body weight. The detailed timeline of behavioral tasks, progressing from place-action to object location memory (OLM), object recognition memory (ORM), and finally to open field and elevated plus maze (EPM) tests, is provided in Supplementary Figure 4.

##### Open field

An automated video system (Video-Mot II, TSE, Bad Homburg, Germany) recorded the locomotion test in a square plexiglass test chamber measuring 40 cm × 40 cm × 50 cm. The measurement was carried out for 10 minutes and the distance traveled was recorded. The center zone was determined as a 25.8 cm × 25.8 cm square located in the center of the box. The open arena is cleaned with distilled water and 70% ethanol after each mouse.

##### EPM

The elevated plus-maze used in the experiment comprised two open arms (25 cm × 5 cm) and two closed arms (25 cm × 5 cm × 15 cm) connected via a central platform (5 cm × 5 cm) and was 50 cm above the ground. The mice were placed on the central platform facing an open arm and allowed to explore for 5 minutes. The time spent and number of entries made in each arm and the central zone were recorded. An entry was defined as all four paws of the mouse entering a new compartment. The plus-maze is cleaned with distilled water and 70% ethanol after each mouse.

##### OLM test

Mice were studied in a rectangular arena (23 cm × 30 cm), which had different visual cues on each wall. Before the actual experiment, the mice were habituated in the experimental room for 30 minutes. During the five-day habituation phase, the mice were placed in the middle of the arena to explore for 10 minutes. In the training phase, the mice received either saline or CNO treatment 30 minutes before being placed in the arena. Two identical objects were placed in the arena for the mice to explore for ten minutes, and the objects were cleaned with 70% ethanol and distilled water between sessions with each mouse. The mice were tested 24 hours later in the same arena, with one of the objects being moved to a novel location while the other object remained in the same location as in training. The testing phase lasted for ten minutes.

##### ORM test

The ORM test followed a similar protocol as OLM, except for the testing phase. Rather than relocating one of the original objects, the testing phase of the ORM test involved substituting one of the original objects with a novel object. Distinct object sets were employed for the OLM and ORM tests.

##### Quantification of mouse object exploration in ORM and OLM testing

Only mice that met the criteria of having explored the objects for more than 3 seconds during both the training and testing sessions were included in the data analysis. The mouse’s interaction/exploration time with the object is measured when its nose is within 1 cm of the object and oriented directly towards it, ensuring that a line extending from its eyes to nose would intersect the object. However, the following behaviors were not considered part of the exploration time: (1) instances where the mouse did not approach the object (e.g., when the mouse reoriented itself and its nose accidentally came close to the object); (2) instances where the mouse climbed on top of the object (even if it was looking down at the object); (3) instances where the mouse reared on the object but looked over it (e.g., looking at the ceiling); (4) instances where the mouse engaged in repetitive behaviors (such as digging near the object or biting it). The diameter of the object was approximately 2.5 cm, allowing for the calculation of a 1 cm distance relative to the object. During quantification, the experimenter drew a circle around each object to define this 1 cm barrier. The video analysis was blinded, with each original recording video labeled only by a numerical identifier without additional group information for the mice. During the testing sessions, the DI was calculated based on the time spent exploring the moved and unmoved objects, as well as the familiar and novel objects. Specifically, the DI was calculated using either (T_moved_ - T_unmoved_) / (T_moved_ + T_unmoved_) or (T_novel_ - T_familiar_) / (T_novel_ + T_familiar_). The criteria for these calculations were based on previous publications [33].

##### Cross maze task (Place-action test)

The maze test took place in a single room visually divided into two by differences in distal visual cues to define Context A and Context B. The walls in the Context A portion of the room was primarily white in background color and had different patterns on each wall to aid the mice in orientation and localization. Four locations on the floor were marked in Context A, labeled A1, A2, A3, and A4. The walls in the Context B portion of the room were draped in black and had different patterns on them. The cross-maze locations in Context A were mirrored in Context B and labeled B1, B2, B3, and B4. A cross-maze with four identical arms was used in this test, with each arm having 1 cm tall edges which allowed the mice to see the surrounding distal cues and distinguish between the two different environmental contexts. The walls of the maze were made of clear acrylic plastic, and the bottom was made of white acrylic plastic. The maze was placed on a mobile table 1 meter off the floor, which could be moved to the different marked locations in the room. Each arm of the maze was equipped with an automatic water dispenser capable of delivering a 10 µL water reward. The water dispenser was remotely controlled by experimenter, who activated it by pressing a button to release the water before placing the animal in the maze. To minimize the interference with mouse decision-making behavior, the experimenter consistently stood in the same location, positioned in the middle of the room and 1 meter away from the maze.

The Place Action Test is designed to test an animal’s ability to associate left or right turning actions with specific context regardless of the spatial locations within those contexts. The mice must learn to make a left turn when the maze is in any of the locations in context A and a right turn when the maze is in context B. Furthermore, the maze has two starting locations, named north and south, both of which demand the same action at the intersection (left versus right) depending on the Context A or Context B location of the maze. After making a left or right turn, the mice are returned to their home cage for a 1-2 second waiting period before starting a new trial. The animals were tested on maze locations A1, A2, B1, and B2 on a daily basis. Each day consisted of 8 sessions with each session comprised of 7 trials. Successful performance in this task requires the learning of a rule-based cognitive partitioning of the environment, also known as allocentric space.

Mice were handled for five minutes per day for four consecutive days. Habituation on the cross maze followed over the next six days, during which the mice were weighed and injected with a needle to prepare for testing. Each mouse was allowed to explore the maze for 10-15 minutes, during which water droplets were placed on all arms of the maze. The mice were released from either the North or South arm and allowed to follow the water to explore the entire maze before being returned to a resting cage while the water droplets were replenished. Each mouse underwent four of these sessions per day, alternating between being released from the North and South arms. Initially, this was done only in one of the marked locations in the first four days, but later two days alternated between two locations within the room. All habituation and training occurred exclusively within the A1, A2, B1, and B2 locations.

Following the habituation period, the mice underwent a six-day per week training phase. On the sixth day of the week, enough water was provided to ensure the mice maintained a healthy weight (at least 85% of their original body weight). During the training phase, each mouse underwent eight sessions per day, one for each of the eight combinations of four locations (A1, A2, B1, B2) and two releasing arms (North and South). To prevent memorization of a fixed sequence of left and right turns, the order of the sessions was randomized each day. Prior to each training session, mice were weighed and given a simulated injection. Each session consisted of the placement of a 10 µL water reward at the end of the target arm before the mouse was released at the end of either the North or South arm. Regardless of which arm they explored, the mouse was removed from the maze and placed back into the resting cage after reaching the end of an arm. Each session was repeated at least seven times and up to 12 times if the mouse struggled to consistently reach the correct arm. Any sessions with particularly poor accuracy or failed first trials were repeated after the eight sessions. Following one week, the training was fixed to 7 trials and 8 sessions per day for each mouse. The training period lasted between 15 to 31 days, depending on the length of time required for each mouse to consistently reach the correct arm with at least 80% accuracy overall and 100% accuracy on the first trial of each session.

To ensure the accuracy and reliability of our findings, we conducted both familiar location testing (Test 1) and novel location testing (Test 2) twice. Each phase spanned four days, during which mice were weighed and given an injection of either saline or CNO 30-40 minutes before being tested. Every other day, the mice underwent seven trials for each of the eight combinations of releasing arm and maze locations. The testing procedure was similar to that of the training phase, with the exception that no additional trials or sessions were conducted for poor performance. Following the familiar location testing, the novel location testing phase began and lasted for four days. The testing conditions were kept the same as those of the familiar location test, except that the maze was placed in novel locations A3, A4, B3, and B4 in the behavior room. These locations were not used during the habituation or training phases, which allowed us to assess the generalizability of learning across the spaces of Context A and Context B.

The percent accuracy for each training or testing day was calculated using the following formulas: accuracy per day, accuracy at context A, accuracy at context B, accuracy at context A with North releasing location, accuracy at context A with South releasing location, accuracy at context B with North releasing location, and accuracy at context B with South releasing location. These percentages were calculated for every day of training or testing. To compute the percentage accuracy, the formula below was used: Percentage accuracy = (number of correct trials / total number of trials) x 100%.

### Data quantification

To obtain high-resolution images of labeled cells in brain sections, we used an automated slide scanning acquisition software (Olympus VS-ASW 2.9.2, Evident Scientific, MA, USA) with a VS-BX Olympus microscope and a high-sensitivity Hamamatsu ORCA-Rash4.0 camera. We also utilized a confocal microscope (Fluoview 3000, Olympus Life Science Microscopy, MA, USA) with z-stack and tile scanning features under 20X objectives with varies magnification 1x, 2x and 3x for imaging labeled cells in selected sections. The image acquisition was performed using the Fluoview software. During 20x imaging, the emission signal for data acquisition by 640-nm excitation was acquired from 650 nm to 750 nm (pinhole: 1 AU). For data acquisition by 561 nm excitation, the emission was acquired from 570 nm to 620 nm (pinhole: 1 AU). For data acquisition by 488 nm excitation, the emission was acquired from 500 nm to 540 nm (pinhole: 1 AU). For data acquisition by 405 nm excitation, the emission was acquired from 430 nm to 470 nm (pinhole: 1 AU). The acquired image stack had a thickness of about 15 μm per brain section with a step size of 2 μm. The OIR file format was used for image acquisition, which was then converted to a maximal projection image in a TIFF format. To ensure complete and unbiased analyses of virally labeled neurons across the series of brain sections, quantitative examinations were conducted using Adobe Photoshop software (Adobe Systems, San Jose, California, USA).

In our rabies tracing experiments, we followed the established counting protocol as described in our previous publication [34]. Firstly, we selected the brain section containing the target region, RSC, to identify EGFP and DsRed doubled-labeled starter neurons that are restricted to RSC granular area. Cases where starter neurons leak into other RSC subregions or surrounding regions, such as cingulate cortex or CA1, are excluded. The starter cells were manually counted using the counting tool in Photoshop. Subsequently, we aligned the remaining viral-infected brain sections with Franklin and Paxinos’ mouse brain atlas images [35] to identify the anatomical structures for the quantification of labeled cells in specified brain regions. It is important to note that we did not use any stereological protocol, and all labeled cells in each section of the brain section series (i.e., 1 out of every 3 sections) were counted to examine the virally labeled neurons in different brain structures. For quantification, we operationally defined an input CSI as the ratio of the number of presynaptic neurons in a brain region of interest (e.g., DS) versus the number of postsynaptic (starter) neurons in RSC. Additionally, we calculated the PI as the ratio of the number of presynaptic inputs versus the total number of inputs. Both CSI and PI were used to analyze the input regions and showed similar trends.

To address the degree of overlap, or colocalization, of M2-projecting and AD-projecting RSC populations, we adhered to the same protocol employed for rabies-labeled neuronal counting. We manually quantified the AAV-GFP and AAV-mRuby labeled presynaptic input neurons and the DAPI-stained cells within RSCg subregion. The percentage of the fluorescence-labeled neurons was determined by dividing the count of fluorescence-labeled neurons by the total number of DAPI-stained cells in the RSCg region. To evaluate random colocalization, we generated 1000 random assignments of DAPI-labeled RSCg neurons as M2- and AD-projecting RSCg neurons according to the observed proportions of M2 and AD projecting neurons found in our actual data.

In the anterograde AAV-synaptoTAG2 tracing experiment, the normalized fluorescence density was measured using Photoshop software. The integrated intensity and area of the selected region of interest (ROI) on one brain section were quantified, with baseline fluorescence levels determined from three random areas devoid of fluorescent signals within the same brain section. Normalized fluorescence intensity was computed by dividing the integrated density of each region by its corresponding area and then subtracting the baseline intensity.

### Statistics

The data are reported as the mean ± SE, and statistical analysis conducted using GraphPad Prism software (San Diego, CA, USA) and MATLAB. For statistical comparisons of tracing data between groups, we used the Mann–Whitney U test, followed by False Discovery Rate (FDR) adjustment for multiple comparisons. This test is appropriate for a relatively small sample size and does not require assumptions of normality or equal variance needed for parametric tests. An FDR-adjusted q-value ≤ 0.05 was considered statistically significant. In the cross-maze test, we used the Wilcoxon signed rank test, which calculates the p-value of a paired, two-sided test.

## Results

### M2- and AD- projecting neurons constitute different populations of RSCg neurons

**Fig. 1:**
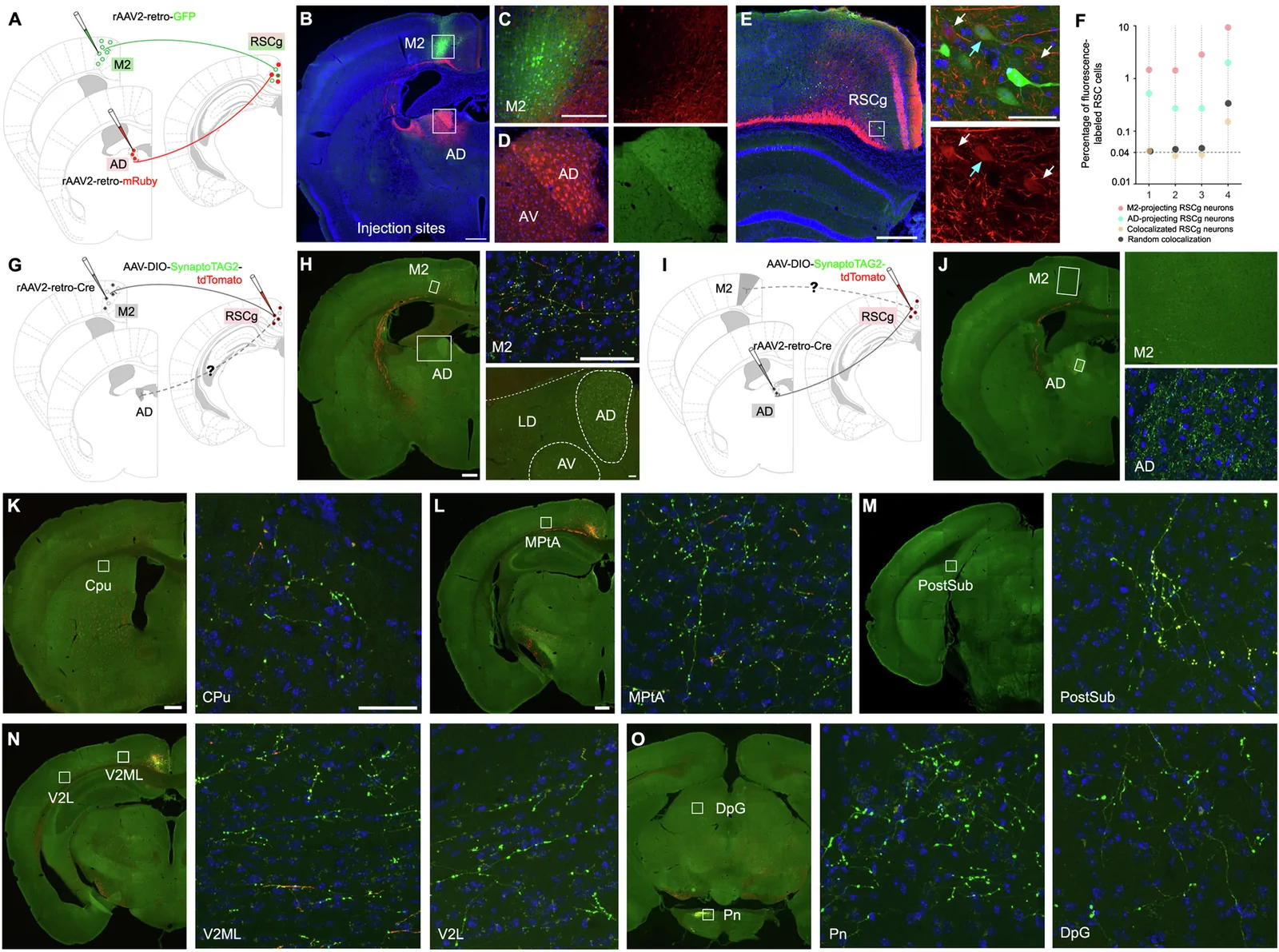
Distinct RSC neuronal circuits corresponding to M2- and AD-projection neurons revealed by retrograde adeno-associated (rAAV2-retro) virus and anterograde AAV-SynaptoTAG2 virus. **A** The schematic illustrates the use of retrograde AAV viral tracers to retrogradely trace M2- and AD-projecting neurons to RSC subregions. rAAV2-retro-GFP and rAAV2-retro-mRuby were injected into M2 and AD, respectively, in C57 mice. The viral expression and retrogradely traced input neurons were visualized using GFP and mRuby fluorescence. **B**–**D** Coronal example of injection sites in M2 (**C**) and AD (**D**). DAPI staining is blue. **E** Images show the distribution of M2- and AD-projecting neurons in RSC granular area (RSCg) with higher-resolution images in right panels. The white box frames the region with co-localizing neurons in RSCg. The blue arrow points to the co-localizing neurons that express both GFP and mRuby. The white arrows indicate AD-projecting RSCg neurons, labeled by mRuby. DAPI staining is blue. To visualize cell bodies, a 30 μm thick brain section was imaged using 20x confocal microscopy with a 2 μm z-stack step size, and then converted into 10- μm range images, as the signal of AD-projecting neurons is often obscured by axons and dendrites. The scale bar (400 μm) applies to left panels in **A–E**. The scale bar (40 μm) applies to all the magnified higher-resolution panels. **F** A quantitative analysis of the degree of M2- and AD-projecting RSCg neurons. The percentage of fluorescence-labeled cells was calculated by dividing GFP-, mRuby or GFP and mRuby fluorescence-labeled neurons by the total number of DAPI-stained cells in the RSCg region. Each pink, green and yellow circle represents the mean percent labeling of M2-projecting, AD-projecting and colocalized RSCg neurons, respectively, in an individual mouse. Random colocalization was determined through 1000 iterations of randomly assigning all M2-projecting and AD-projecting neurons in a given slice to any DAPI positive cells in the same slice. The individual black dot represents the 95^th^ percentile range for 1000 iterations of randomized colocalization. N = 4 mice. Data were quantified from 4 RSC sections, sampled from 8 out of 24 wells, with a 90 μm distance from each well. **G** Schematic of anterograde tracing of synaptic connections of M2-projecting RSCg neurons using the dual injection approach. The synapoTAG2 AAV (AAV-DIO-synaptoTAG2-GFP/tdTomato) is designed with bicistronic expression of soluble tdTomato and a presynaptic EGFP-synaptobrevin-2 fusion protein driven by the synapsin promoter in the AAV. The rAAV2-retro-Cre injection in M2 retrogradely labels M2-projecting RSC neurons with Cre; then the Cre dependent AAV-synaptoTAG2 is injected into RSCg to trace synaptic connections in C57 mice. **H** Mapping of the projection regions in M2 and of M2-projecting RSCg neurons. **I**, **J** are formatted in the same way as **G–H** to show the projections of AD-projecting RSCg neurons. **K**–**O** Shared collateral projection regions of M2- and AD-projecting RSCg neurons, projection regions for both populations include the caudate putamen (CPu), parietal cortex, medial (MPtA) and lateral (LPtA), postsubiculum (PostSub), secondary visual cortex, medial and lateral subregions (V2ML and V2L), pontine nuclei (Pn) and superior colliculus (DpG). The panels are arranged by low-resolution (10x, left panel) and higher resolution 20× images (right panels). The red tdTomato labeling marks axonal fibers, while green EGFP-labeling marks the synaptic terminals projecting from the M2-projecting neurons. The yellow overlap of red and green labeling indicates the presence of labeled synaptic connections. The data presented here are from M2-projecting mice (AD-projecting results can be found in Fig. S1). The scale bar (400 μm) applies to left panels in H-O. The scale bar (40 μm) applies to all higher-resolution magnified panels.

To determine whether the M2- and AD-projecting neurons are distinct, but co-localized populations in RSC, we used a designer AAV variant (rAAV2-retro) to retrogradely trace RSC neurons with efferents to these regions (Fig. 1). We injected rAAV2-retro-GFP and rAAV2-retro-mRuby into M2 and AD, respectively, in C57BL/6 mice (Fig. 1A). The GFP and mRuby fluorescent proteins allowed us to visualize the viral expression in the two injection sites, as well as the retrogradely traced M2- and AD-projecting neuron populations in RSC (Fig. 1B–E). Most neurons were found in the anterior and mid regions of the RSC. The mean coverage of the M2 injection site was approximately 7.11%, while the mean coverage of AD was 33% (n = 4 mice, Figure S1K and Table S2). While the M2 injections yielded a smaller coverage size, we nevertheless found a higher percentage of projection-specific RSC neurons (3.17%) compared to AD neurons projecting to the RSC region (0.63%) (Figure S1K).

The degree of colocalization among AD-projecting and M2-projecting RSC neurons was examined by calculating the percentages of AD-projecting, M2-projecting, and AD- and M2-projecting neurons relative to the total number of RSC cells in a given area (the latter defined by analysis of percentage-stained slices). The raw counting data highlight a large difference in the distribution of projection-specific neurons and colocalized neurons, showing that the observed colocalized neurons projecting to both AD and M2 are 54 times fewer than those projecting solely to M2 and 9 times fewer than those projecting solely to AD. To contextualize these findings, we performed a randomization analysis wherein the “M2-projecting” and “AD-projecting” assignments of neurons were randomized across the total population of RSC neurons in a given slice. For each of 1000 iterations of this process, the number of neurons assigned as both “M2-projecting” and “AD-projecting” was determined in order to estimate the range of colocalized neurons expected by chance. Quantitative analysis revealed that the actual, or observed, colocalization (OC) for all four animals fell within the 95^th^ percentile range for 1000 iterations of randomized colocalization (RC, Fig. 1F). This indicates that the very low percentage of RSC neurons with projections to both AD and M2 and follows the percentage expected by chance.

Next, we utilized a combinatorial approach using rAAV2-retro-Cre and AAV-DIO-synaptoTAG2-GFP/tdTomato viruses to examine the collateral projections of M2- and AD-projecting RSC neurons and map the brain-wide circuit outputs from these RSC subpopulations. We injected rAAV2-retro-Cre into M2 or AD in C57BL/6 mice, followed by the injection of Cre-dependent AAV-DIO-synaptoTAG2-GFP/tdTomato in RSC (n = 3 mice per group) (Fig. 1G, I). The AAV-DIO-synaptoTAG2 virus is engineered to express EGFP-fused synaptobrevin-2 protein in synaptic terminals as well as cytoplasmic tomato under control of Cre-recombinase [31]. Using this approach, the AAV- synaptoTAG2 infected neurons are filled with tdTomato including axon fibers and their synaptic terminals are labeled by GFP in efferent projection regions.

Immunostaining analysis was performed on sections from M2 and AD regions using a Cre antibody to confirm the restricted localization of the retrograde rAAV2-retro-Cre virus within the target regions (Fig. S1A, C). M2- and AD-projecting neurons, labeled with GFP and tdTomato, respectively, were restricted to the granular region of RSC, as illustrated in Fig. S1B and D. By this method, we found that M2- and AD-projecting RSCg neurons exhibit no reciprocal projections between M2 and AD. That is, we did not find observable synaptic terminals from M2-projecting RSC neurons in AD (Fig. 1H) and did not find observable synaptic terminals in M2 from AD-projecting RSCg neurons (Fig. 1J). Our findings are supported by quantifying the fluorescence intensity of synaptic terminals. The M2-projecting RSC group exhibits greater intensity in M2 (M2 = 9.10 ± 0.24 A.U.; AD = 0.31 ± 0.06 A.U.; n = 2 cases, 3 slices per brain region) while the AD-projecting RSC group displays higher intensity in the AD region (M2 = 0.25 ± 0.05 A.U.; AD = 11.83 ± 1.32 A.U.; n = 2 cases, 3 slices per brain region) (Fig. S1J and Table S2). However, M2-projecting and AD-projecting RSCg neurons do share collateral projections to other brain regions, including the parietal cortex and sensory visual cortex, with projections located in layer 6 (Figs. 1L and N and S1). EGFP expression resulting from both AD- and M2-projecting RSC populations was also found in other sensorimotor-controlled brain regions, with strong expression in pontine nuclei for both AD-projecting and M2-projecting neurons, and weaker expression in superior colliculus and postsubiculum (Figs. 1M and O and S1).

Thus, both by examining overlap between retrogradely labeled RSC neurons projecting to AD versus M2 and by visualizing overlap, or lack thereof, in target brain regions of RSCg neurons, we find little overlap between M2-projecting and AD-projecting RSCg neuron sub-populations. We next examined similarities and differences in the strength of inputs to these neuron populations across the wide range of brain regions providing afferents to RSCg.

### Utilization of monosynaptic retrograde rabies virus to map brain-wide inputs to RSC subpopulations

**Fig. 2:**
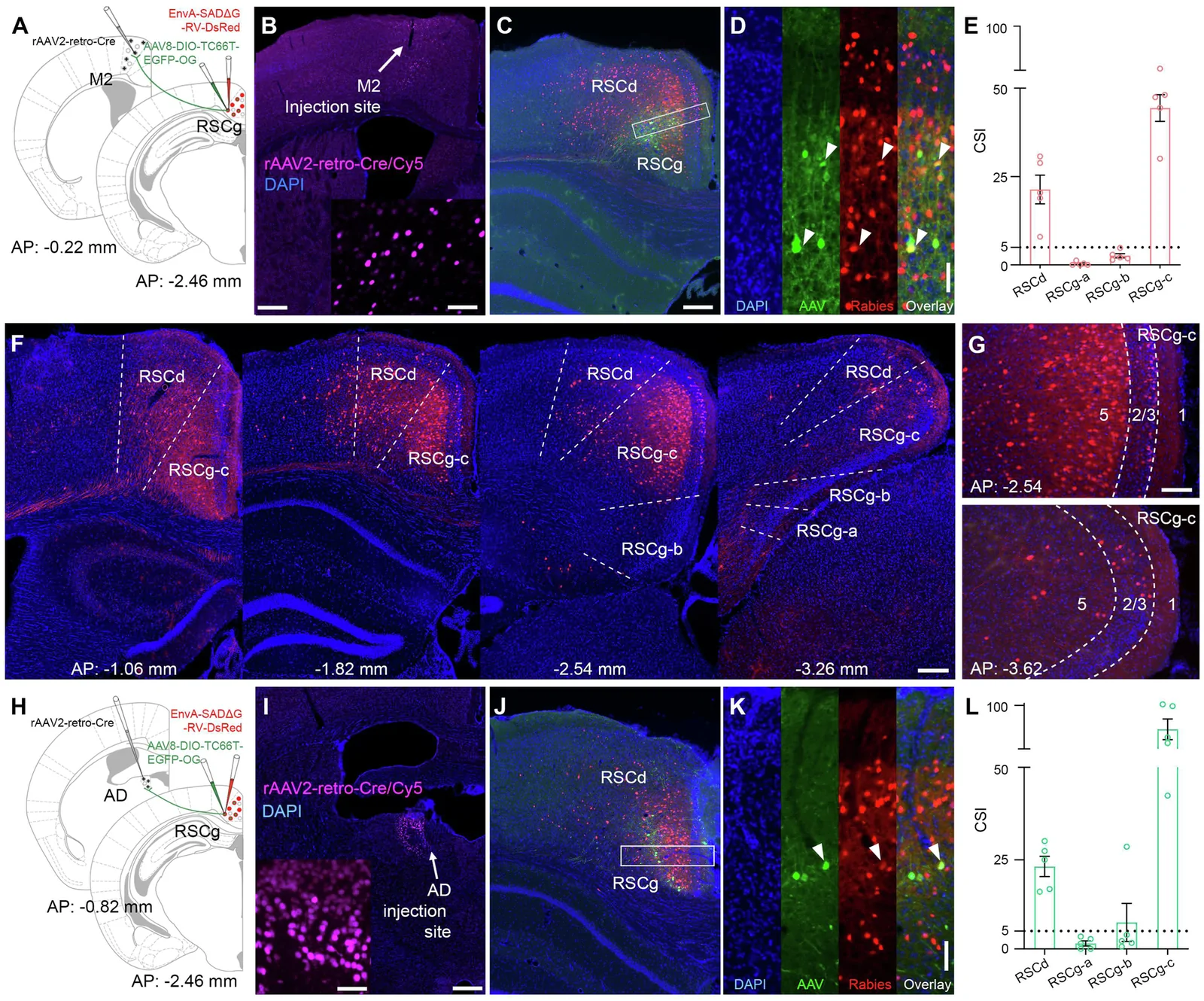
Monosynaptic rabies viral tracing reveals RSC intrinsic inputs to M2-projecting and AD-projecting RSCg neurons in C57BL/6 mice. **A** Schematic of monosynaptic retrograde rabies tracing (RV) of synaptic inputs to M2-projecting RSCg cells. The rAAV2-retro-Cre injection in M2 retrogradely drives Cre expression in M2-projecting RSC; then the Cre dependent AAV helper virus (AAV8-hSyn-DIO-TC66T-2A-EGFP-2A-OG) is injected into RSCg, followed by injection of an EnvA-pseudotyped SAD ΔG rabies virus three weeks later. **B** Immunostaining and microscopy images depict Cre positive neurons in the M2 injection site. rAAV2-retro-Cre labeled neurons are stained by a Cy5-conjugated secondary antibody and visualized as pink in the enlarged inset. **C, D** Coronal sections of injection in RSCg. Injection sites are shown in white boxes (**C**) with magnified images in (**D**). EGFP-labeled AAV-helper virus expressed in M2-projecting Cre+ neurons. Rabies is labeled by DsRed, DAPI is blue. White arrowheads indicate EGFP and DsRed double-labeled starter neurons. **E** Quantitative mapping of the connection strength of the intrinsic RSC subregion inputs to M2-projetcing RSCg neurons. Connection strength index (CSI) is calculated by the number of presynaptic input neurons divided by the number of starter neurons in RSCg. The individual circle represents one case (n = 5). Data are measured from M2-projecting RSCg neurons (N  =  5 cases) and AD-projecting RSCg neurons (N  =  5 cases) and are presented as the mean ± s.e.m. **F**, **G** Example of input- mapped local RSC subregions along the longitudinal axis. AP number indicates the distance relative to bregma. **G** represents magnified views corresponding to the panels in (**F**), indicating that the intrinsic connections vary across different layers along the anterior/posterior axis. **H**–**L** follow the same order as (**A**)–(**E**) and reveal RSC subregions inputs to AD-projecting RSCg neurons.

To investigate the brain-wide afferent distributions of projection-specific RSCg neuron sub-populations, we used a Cre-dependent monosynaptic retrograde rabies tracing system in conjunction with Cre-expressing retrograde AAV injection in targeted brain regions in C57BL/6 mice. We targeted M2-projecting or AD-projecting neurons in RSCg using rAAV2-retro-Cre for monosynaptic rabies tracing from Cre positive neurons in RSCg (Fig. 2).

**Fig. 3:**
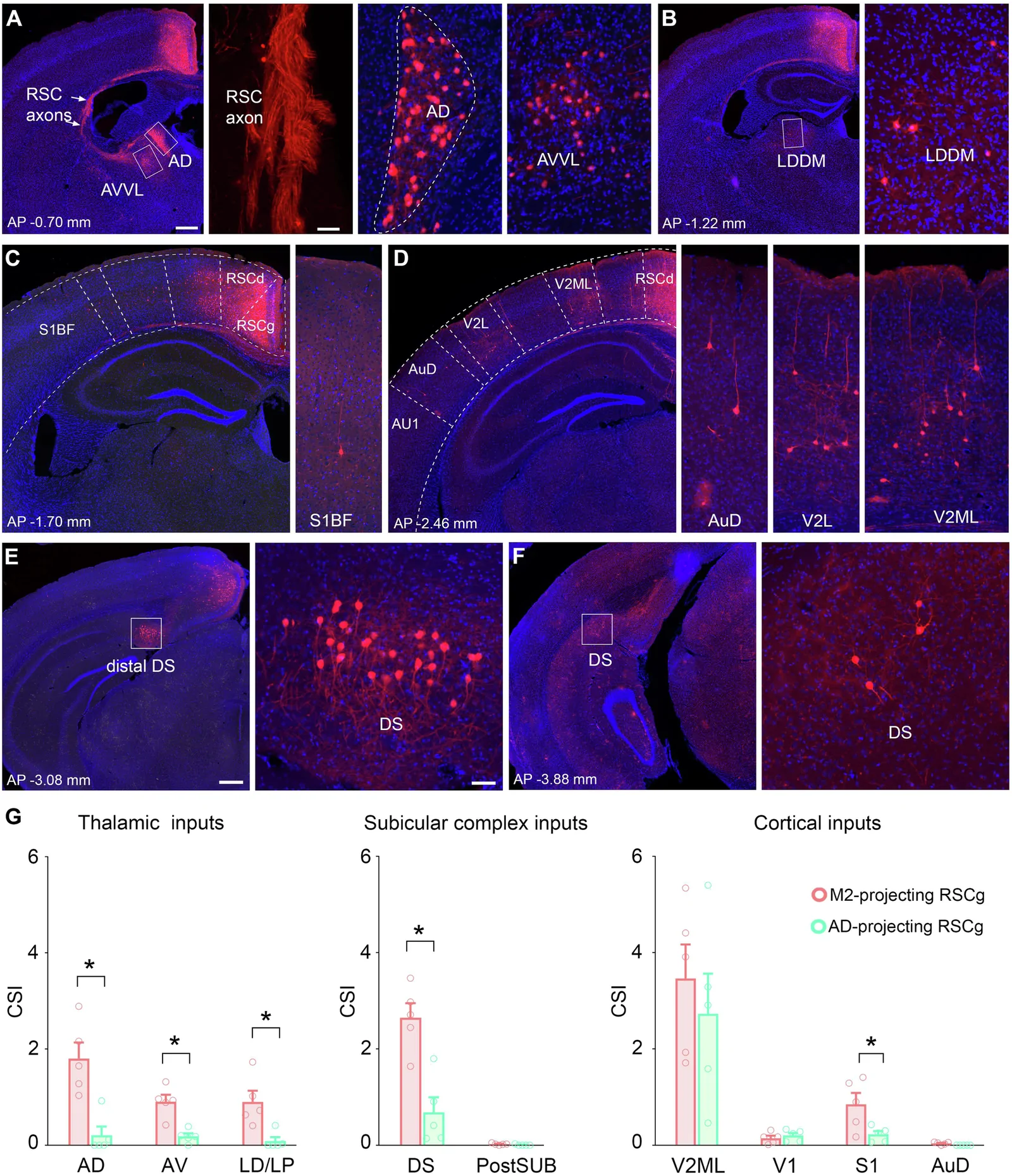
Monosynaptic rabies viral tracing reveals stronger thalamocortical and subicular inputs onto M2-projecting RSCg neurons compared to AD-projecting RSCg neurons. **A**–**F** Representative images show presynaptic input regions that have stronger connections with M2-projecting RSCg neurons than AD-projecting RSCg neurons. Input-mapped neurons in the anterior thalamus (**A**), lateral thalamus (**B**), sensory cortex (**C**, **D**) and subiculum (**E**, **F**) are shown (See Fig. S2 for more input regions). The expression of presynaptic RV is visualized with DsRed in red, DAPI staining is blue. The AP number indicates the distance from coronal sections (30- μm thick) relative to the bregma. The scale bar (400 μm) applies to left panels in A-F, while the scale bar (50 μm) applies to all the higher-resolution magnified panels. **G** Quantitative analysis of input connection strengths of M2-projecting and AD-projecting RSCg neurons, showing the CSI for each input-mapped brain structure. Data are measured from M2-projecting RSCg group (N  =  5 cases) and AD-projecting RSCg group (N  =  5 cases) and are presented as the mean ± s.e.m. The significance of differences for each input region was tested using the Mann-Whitney U test and adjusted for multiple comparisons using the FDR method, with statistical significance set at *P  <  0.05. M2, secondary motor cortex; RSCd, dysgranular layer of retrosplenial cortex; AD, anterodorsal thalamic nucleus; AVVL, anteroventral thalamic nucleus; LDDM, laterodorsal thalamic nucleus; SIBF, primary somatosensory cortex, barrel field; MPtA, medial parietal association cortex; V2L and V2ML, secondary visual cortex, lateral and mediolateral areas; AuD, primary and secondary auditory cortex; DS, dorsal subiculum. Further details for the data are provided in Supplementary Table 2.

**Fig. 4:**
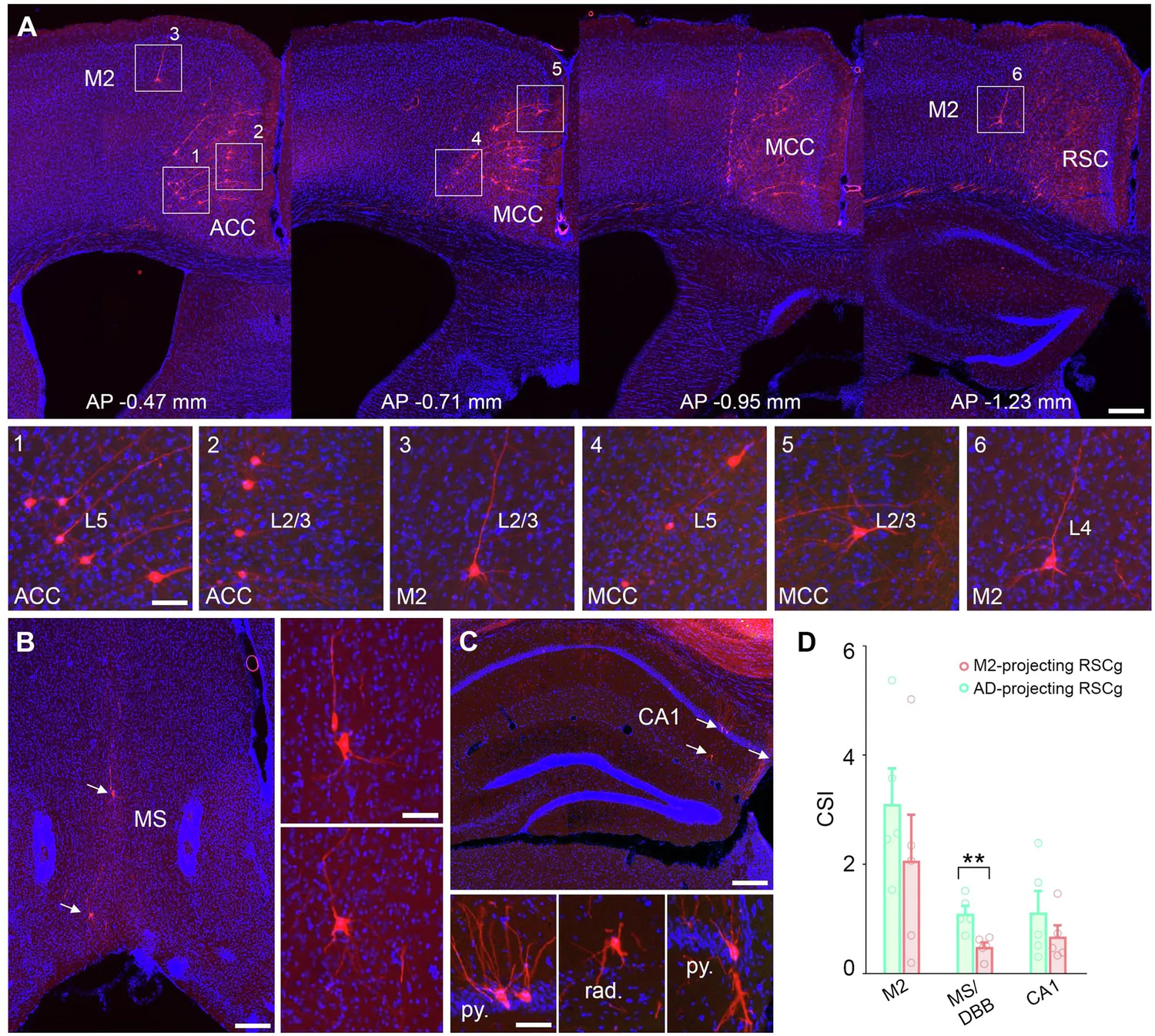
The medial septal region shows greater inputs to AD-projecting RSCg neurons than to M2-projecting RSCg neurons. **A** Representative images show the distribution of cingulate cortex and M2 inputs to AD-projecting RSCg neurons. The expression of presynaptic RV is visualized with DsRed in red, DAPI staining is shown in blue. The AP number indicates the distance from the coronal section (30- μm thick) relative to the bregma. **B**, **C** Coronal images show presynaptic inputs in MS and CA1 (See Fig. S1 for more input regions). The scale bar (400 μm) applies to the top panels in (**A**) and (**C**). The scale bar (200 μm) applies to the left panel in (**B**), and the scale bar (50 μm) applies to all higher resolution magnified panels. **D** Quantitative analysis of input connection strengths of M2-projecting and AD-projecting RSCg neurons show the CSI for each input-mapped brain structure. Data are measured from M2-projecting RSCg neurons (N  =  5 mice) and AD-projecting RSCg neurons (N  =  5 mice) and presented as the mean ± s.e.m. The significance of differences for each input region was tested using the Mann-Whitney U test, and adjusted for multiple comparisons using the FDR method, with statistical significance set at *p  <  0.05. ACC, anterior cingulate cortex; MCC, medial cingulate cortex; MS, medial septal nucleus; M2, secondary motor cortex. Detailed data are provided in Supplementary Table 2.

The rabies tracing system targets specific cell types using EnvA pseudotyping and limits transsynaptic spread to direct presynaptic inputs using glycoprotein gene-deleted (ΔG) rabies virus and trans-complementation [32, 36, 37]. Specifically, the ΔG rabies virus (deletion mutant, SAD-B19 strain) is pseudotyped with the avian sarcoma leucosis virus glycoprotein EnvA (EnvA-RVΔG -DsRed), which can only infect neurons that express the avian tumor virus receptor A (TVA). The TVA is an avian receptor protein that is absent in mammalian cells unless it is expressed through exogenous gene delivery. The helper AAV (AAV8-DIO-TC66T-oG-EGFP) delivers the rabies glycoprotein (RG) and TVA genes to Cre-expressing neurons in specific injection sites, such as RSCg, in the present study. With the combination of retrograde rAAV2-retro-Cre injection in M2 or AD, we were able to label M2 or AD projection-specific RSCg neuron sub-populations with Cre. The deletion-mutant rabies virus can then be trans-complemented with the expression of rabies glycoprotein (RG) in the same TVA-expressing cells to enable its retrograde spread restricted to direct presynaptic neurons. Because these presynaptic neurons lack RG expression, the virus cannot spread further beyond these cells. Our rabies virus labeling technique allows for identification of starter cells at the injection site, and permits the quantitative measurement of connectivity strengths of input mapped brain regions. The starter cells are unambiguously identified by their EGFP and DsRed expression from the helper AAV and RVΔG-DsRed rabies genomes, respectively (Fig. 2D, K). We saw robust expression of DsRed in presynaptic neurons to projection-specific RSC neurons in the input-mapped brain regions (Figs. 2–4). We calculated the connection strength index (CSI) as the ratio of the number of presynaptic neurons in a brain region of interest to the number of postsynaptic (starter) neurons in RSCg (Fig. S2).

### Local connectivity of M2- and AD-projecting RSCg sub-populations

We virally traced circuit connections to M2-projecting and AD-projecting starter cells located in RSCg subregion c (RSCg-c). To accurately define the coordinates for the RSC injection region, we relied on AAV retrograde tracing results with GFP and mRuby fluorescent labeling (Fig. 1E). This approach allowed for precise localization of the starter neurons within RSC, granular area while avoiding leakage into the anterior cingulate cortex. The injection site is within the deep layer of RSCg-c, as shown in Fig. 2C and J. The number of starter neurons in the M2-projecting group is relatively higher compared to the AD-projecting group (average number of starters per case: M2-projecting group, 113 ± 17 neurons, n = 5 mice; AD-projecting group, 27 ± 7 neurons, n = 5 mice) (Table S2). The starter cells are clearly identified by their EGFP and DsRed expression (Figs. 2D, K). We also performed immunostaining of M2 and AD sections with a Cre antibody to confirm that the retrograde rAAV2-retro-Cre virus was restricted to target regions (Fig. 2B, I). We first examined the local synaptic connections in the subregions of RSC along the longitudinal axis. Our findings reveal that both M2- and AD-projecting RSC neurons establish connections with all RSC subregions (Fig. 2E, L), with the connection strength being relatively higher in the AD-projecting neurons compared to the M2-projecting neurons (CSI for RSCg: M2 = 49.596 ± 6.810, AD = 85.467 ± 14.945, *p* = 0.0159, q = 0.140; CSI for RSCd inputs: M2 = 24.162 ± 4.343, AD = 23.861 ± 2.436, *p* = 0.056, q = 0.922; Mann-Whitney U test, followed by False discovery rate test; n = 5 per projecting-group) (Fig. S2B and Table S2). The majority of local inputs are concentrated in the RSC, dysgranular area (RSCd) and the granular area (RSCg), subregion c while fewer input neurons are present in RSCg subregions a and b. Along the anterior-posterior (longitudinal) axis, the middle portion region of RSC (AP: -1.82 mm relative to Bregma) exhibits the strongest intrinsic input to the M2-projecting and AD-projecting RSCg populations with a gradual decrease as one moves from anterior RSC (AP: -1 mm relative to Bregma) to posterior RSC (AP: -4mm) along the longitudinal axis (Figs. 2F, S2B). These local inputs to RSCg projection neurons are located in layers 2/3 and 5. More neurons are distributed in the superficial layer of layer 2/3 and these appear to have a relatively smaller soma size (Fig. 2G).

### Differential distributions of afferent inputs to M2- and AD-projecting RSCg subpopulations

Having established that RSCg neurons with projections to M2 and AD are largely separate populations of neighboring RSC neurons, we next sought to distinguish them further by quantifying the strength of afferents each group obtains from among the many and diverse sources of RSC afferents. The combined afferent and efferent connectivity patterns for these two populations effectively define the degree of independence and potential differential function of RSC circuits.

We find that both M2- and AD-projecting pathways have circuit connectivity with overlapping input regions, including medial septal nucleus, cingulate cortex, visual cortex, motor cortex, somatosensory cortex, parietal cortex, subiculum, the hippocampal formation, and multiple thalamic nuclei (Fig. S2). Notably, these extrinsic input regions exhibit smaller connection strength numbers in comparison to RSC intrinsic connections. No significant differences in the inputs originating from the visual cortex, parietal cortex, CA1 and postsubiculum were observed (Fig. S2A).

The boundary between the cingulate cortex and RSC is not well-defined, with different atlases providing varying border lines. Paxinos and Franklin use Cg1/2 to denote cingulate cortex, which extends into RSC at Bregma -0.48 mm [35]. However, the Allen Atlas splits the cingulate cortex into anterior (ACC) and medial (MCC) portions. The ACC terminates at Bregma -0.25 mm, which falls within Cg1/2 range in Paxinos’s atlas. The MCC, on the other hand, spans from Bregma -0.25 mm to -1 mm, encompassing parts of the RSC regions in Paxinos’s atlas. To better depict the cingulate inputs, we have opted to use to draw a distribution of cingulate inputs along anterior-posterior axis relative to Bregma. The data in Fig. S2B indicates that the strength of cingulate cortex and local retrosplenial cortex inputs gradually increases along the anterior-posterior axis, from ACC to MCC and eventually transitioning to the RSC.

In contrast to the similar input strengths from many RSCg sources onto M2-projecting and AD-projecting RSC sub-populations, we also find distinct patterns of input to the pathway-specific RSCg neurons. Specifically, M2-projecting RSCg neurons receive significantly stronger inputs from anterodorsal, anteroventral, and laterodorsal/lateral posterior thalamic nuclei, dorsal subiculum and somatosensory cortex (Fig. 3). The M2-projecting neurons exhibit higher CSI values for inputs arising from dorsal (AD) and ventral (AV) portions of anterior thalamus and from lateral dorsal thalamus (LD) (CSI for AD inputs: M2 = 1.803 ± 0.331, AD = 0.209 ± 0.180; *p* = 0.0079; CSI for AV inputs: M2 = 0.906 ± 0.143, AD = 0.185 ± 0.062; *p* = 0.0079, q = 0.034; CSI for LD inputs: M2 = 0.904 ± 0.229, AD = 0.089 ± 0.081; *p* = 0.0159, q = 0.048; Mann-Whitney U test, followed by False discovery rate test, n = 5 per projecting-group) (Fig. 3A, B, G and Table S2). Dorsal subiculum (DS), a major contributor of inputs to RSC, has 3-fold higher connection strength with M2-projecting RSCg neurons compared to AD-projecting neurons (CSI for DS inputs: M2 = 2.647 ± 0.303, AD = 0.684 ± 0.312; Mann-Whitney U test, *p* = 0.0159, q = 0.048; n = 5 per projecting-group) (Fig. 3E, F, G and Table S2). We also observe significant differences in sensory cortex, including auditory (AuD) and the secondary somatosensory cortex (S1) (CSI for AuD inputs: M2 = 0.041 ± 0.012, AD = 0; Mann-Whitney U test, followed by False discovery rate test, *p* = 0.0476, q = 0.126; CSI for S1 inputs: M2 = 0.853 ± 0.234, AD = 0.245 ± 0.091; Mann-Whitney U test, followed by False discovery rate test, *p* = 0.05, q = 0.034; n = 5 per projecting-group) (Fig. 3C, D, G and Table S2). In the current study, our findings suggest that the auditory cortex projects exclusively to M2-projecting neurons and not to AD-projecting RSCg neurons (Fig. 3G, last panel). Nevertheless, the total number of observed auditory cortex neurons providing inputs to M2-projecting RSC neurons is small. Further work focused on this input to RSC will be needed to determine whether AD-projecting RSC neurons receive no auditory cortex inputs.

AD-projecting neurons receive approximately twice the input from the medial septal-diagonal band (MSDBB) than compared to M2-projecting neurons (Fig. 4B and D, CSI for MSDBB inputs: M2 = 0.488 ± 0.086, AD = 1.099 ± 0.140; Mann-Whitney U test, followed by False discovery rate test, *p* = 0.0079, q = 0.035; n = 5 per projecting-group). Additionally, sparse inputs from distal CA1 pyramidal cell and radiatum layer are shown in Fig. 4C, but no significant difference is observed between the two groups of projection neurons (Fig. 4D). We also note a non-significant trend toward greater input from M2 (CSI for M2 inputs: M2 = 2.066 ± 0.842, AD = 3.102 ± 0.653; Mann-Whitney U test, followed by False discovery rate test, *p* = 0.222, q = 0.471; n = 5 per projecting-group) (Fig. 4A and D). This difference is notable in light of the corresponding difference between M2-projecting and AD-projecting neurons input from AD. The combined data thus indicates that RSC projections to AD and M2 are not characterized by a bias toward reciprocity in the inputs from AD and M2. Rather, AD-projecting neurons gain greater input from M2 and M2-projecting neurons gain greater input from AD.

### Differential contributions of M2- and AD-projecting neurons to spatial cognition

Our circuit mapping data reveal differences in RSC sub-circuits composed of projection-specific RSC sub-populations. AD and DS neurons are well known for their respective tuning to head orientation and location relative to environmental boundaries. The significantly greater input of these afferent sources to M2-projecting RSC neurons is suggestive of potential functional differences in AD- and M2-projecting RSC neurons in spatial cognition. We therefore employed a chemogenetic approach to address the functions of these projection-specific RSC populations in memory for spatial configurations between environmental boundaries and landmarks and for transformation of spatial cognition into action.

**Fig. 5:**
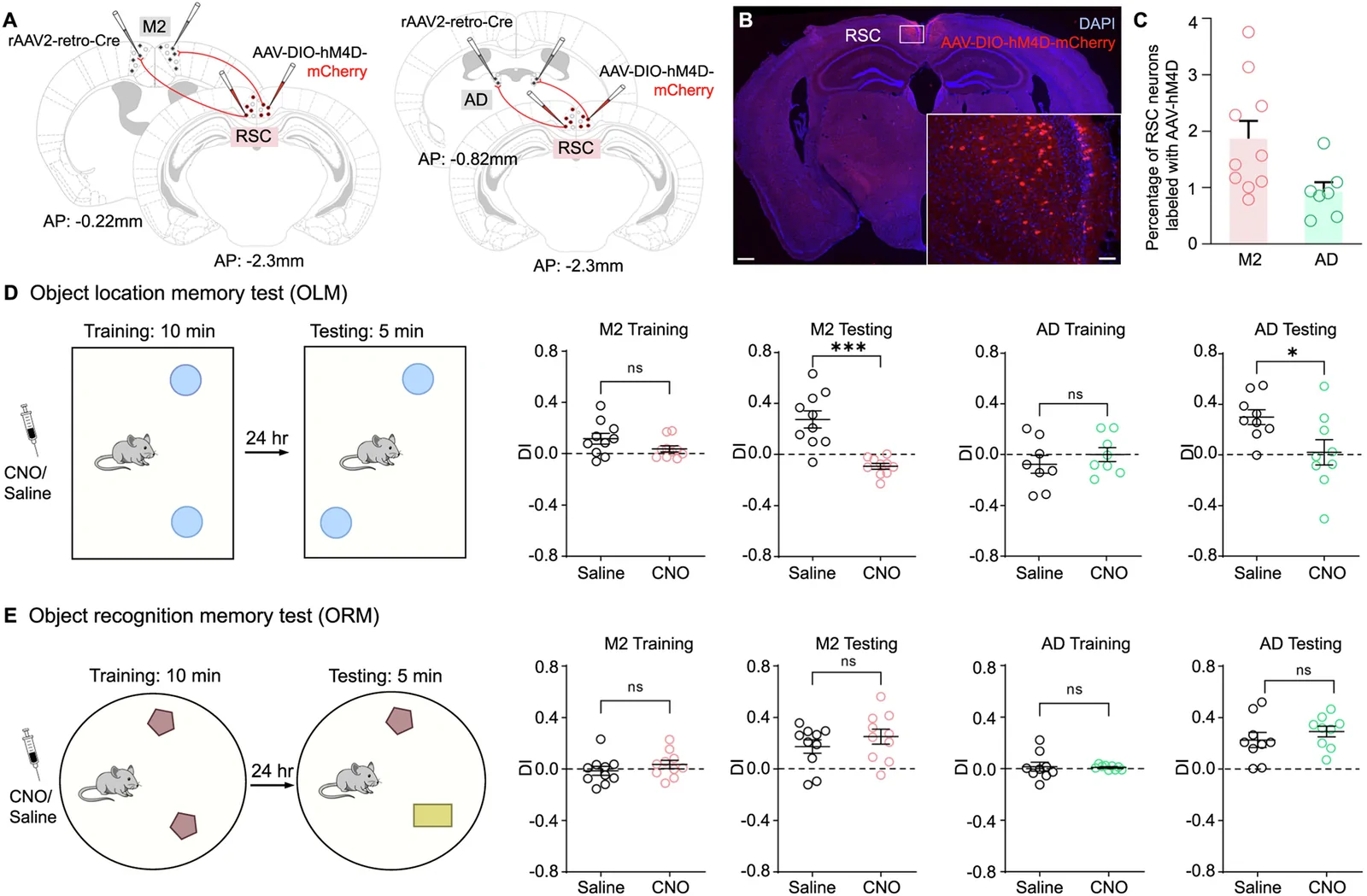
Genetically targeted inactivation of M2- and AD-projecting RSC neurons impair object-related spatial memory but not object recognition memory. **A** Schematic illustration of genetic inactivation of M2-projecting and AD-projecting RSC neurons using dual rAAV2-retro-Cre injection in the M2 (left) or AD (right) and AAV2-DIO-hM4D-mCherry injection in RSC. **B** Histological analysis in coronal brain sections verifies bilateral, spatially restricted hM4D-mCherry expression in the RSC. The bottom right inset shows a higher magnification view of the white square region with hM4D-mCherry-expressing M2-projecting RSC neurons in red. Scale bars, 400 µm and 50 µm (inset). The viral injection experiment was independently repeated in 19 mice (n = 10 in the M2-projecting group; n = 9 mice in the AD-projecting group), with similar results obtained each time. **C** The percentage of hM4D-mCherry labeled RSC neurons in behavioral experiment mice. The percentage is calculated by dividing the number of mCherry-labeled neurons by the DAPI-stained cells in the RSC region. Pink indicates the percentage of M2-projecting RSC neurons. Green represents AD-projecting RSC neurons. Each individual circle represents the mean percent labeling of a group of projection-specific RSC neurons in an individual mouse. N = 10 mice for M2 group, N = 7 mice for AD group. Data were quantified from two RSC sections, sampled from eight out of 24 wells, with a 90 μm distance from each well. **D** Schematic figures for the experimental design and results of the location-related object recognition task (OLM) following CNO-activated inhibition of M2-projecting or AD-projecting RSC neurons during the training session. The box represents the open-field arena, and the blue filled circles indicate the training (left) and test (right) object locations. Before the experiment, mice were handled and habituated to the experimental context in the absence of objects. Mice received a single intraperitoneal injection of control saline or experimental CNO treatment (5 mg per kg) 30 min before the training. The figure depicts the discrimination index (DI) results for the M2-projecting and AD-projecting groups in the OLM task, 24 hours after training. The two middle panels show the DI values for the training and testing sessions in the M2-projecting group, revealing a reduced preference for the moved object in CNO-treated mice compared to saline-treated controls. The two panels on the right show the DI results for the AD-projecting group, indicating no preference for the moved object in CNO-treated mice compared to saline-treated controls. Each data point is represented as a color-coded circle. Black is the saline treated control group, pink is the CNO treated M2-projecting RSC group, and green is CNO treated AD-projecting RSC group. The data is presented as the mean ± s.e.m., with significance denoted by asterisks: *, p < 0.05, ***p < 0.001 for significant differences and ns for nonsignificant differences (Mann-Whitney *U*-test). **E** Schematic illustration of novel object recognition (ORM) testing and the results. In training sessions, mice were trained with two identical objects (brown filled polygon) and tested with one of the original objects replaced with a distinct yellow filled rectangular object. Mice with inhibition of M2-projecting or AD-projecting RSC neurons show comparable preferences for the novel object and these are similar to the saline-treated controls. Each data point is represented as a color-coded circle. Black is the saline treated control group.

Prior literature suggests that extensive lesions of RSC can lead to impairments in object-related spatial memory [38]. In this study, we aimed to investigate the role of projection-specific subpopulations of RSC neurons in spatial cognition. To accomplish this, we employed designer receptors exclusively activated by designer drugs (DREADDs) to inhibit RSC neurons projecting to M2 or AD during behavioral testing in an object location memory task (OLM). As shown in the schematic in Fig. 5A, we selectively expressed hM4D in Cre positive neurons in the M2 or AD by injecting rAAV2-Cre in M2 or AD and AAV2-DIO-hM4D-mCherry in RSC. Cre immunostaining results confirm the restricted localization of rAAV2-retro-Cre virus within the target regions of M2 or AD (Fig. S3E, F). A total of approximately 1.8% of all RSC neurons in M2 animals (n = 10 mice) and 0.9% of all RSC neurons in AD animals (n = 7 mice) were found to express the AAV-hM4D virus (Fig. 5B, C and Table S3d). Subsequently, clozapine N-oxide (CNO) or saline was administered intraperitoneally 30 minutes before the behavioral assessments were made.

The OLM task consists of a training session in which the mice explore an open arena containing two identical objects serving as ‘landmarks’ (Fig. 5D). This is followed by a testing session 24 hours later in the same environment with one of the objects moved to a novel location. We measured the time spent exploring each object in both sessions and calculated a discrimination index (DI) comparing the difference in time spent at the moved object versus the one that remained in the original position. A higher DI reflects better memory retention for the original, now altered, layout of the objects relative to boundaries; a well-trained mouse is expected to spend more time exploring the object placed in a new location during the testing session provided it can remember the two locations presented during the training session. All subjects underwent two trials of the OLM task using different sets of objects with an intertrial interval of 2 weeks.

The results show that inactivating M2-projecting RSC neurons with hM4D/CNO before the training session significantly reduced DI on the testing day; these mice spent similar amounts of time visiting both the stable and moved objects (Fig. 5D and Table S3d). In contrast, the saline control mice discriminated between the stable object from the object whose location had changed, spending significantly different amounts of time exploring each close-up (n = 10 mice per group, saline, 0.274 ± 0.067; CNO, −0.092 ± 0.022; p < 0.0001, Mann–Whitney U test) (Fig. 5D, middle panels). The same pattern of results was found in the AD-projecting RSC control group. Unlike the control mice treated with saline, the CNO treated mice failed to discriminate the moved object on the testing day by spending more time investigating it (n = 9 mice per group, saline, 0.299 ± 0.058; CNO, 0.020 ± 0.099; p = 0.0315, Mann–Whitney U test) (Fig. 5D, right panels). Therefore, our results suggest that both M2 and AD-projecting RSC neurons play a significant role in the development of OLM during the training day.

We also conducted an object recognition memory (ORM) task where we presented two objects during the training session and replaced one of them with a novel object at the same location prior to the test session (Fig. 5E). The recognition of the novel object is also measured by the DI, representing the bias to explore it more extensively than the previously encountered familiar object. We found no significant difference in exploration time between hM4D/CNO-inhibition M2- or AD- groups and the control groups during the testing session (M2: n = 10 mice per group, saline, 0.174 ± 0.053; CNO, 0.252 ± 0.058; p = 0.393. AD: n = 9 mice per group, saline, 0.227 ± 0.059; CNO, 0.292 ± 0.042; p = 0.387. Mann–Whitney U test) (Fig. 5E and Table S3e). All mice exhibited similar exploration time to each object in OLM and ORM tasks. These findings suggest that the encounter and exploration of the objects were balanced between the two groups during the training sessions, indicating normal object-recognition memory. This also indicates that CNO does not affect the general locomotor activity nor the total exploration time of the CNO group relative to controls.

To further understand the two groups of projection-specific RSC neurons, we measured the locomotor activities of mice in an open arena, as illustrated in Fig. S3A. Specifically, we used the Mann-Whitney test to compare the 10-min locomotor activity data of CNO-inhibited M2-projecting RSC mice with that of a saline-treated control group. The results show no significant differences between the two groups in terms of total locomotor movements (Saline, 2678 ± 279.1 cm; CNO, 2366 ± 146.9 cm; p = 0.481; Mann-Whitney test; n = 10 mice per group) and the percentage of distance traveled in the center zone (Saline, 15.4% ± 1.6; CNO, 15.2% ± 1.8; p = 0.971; Mann-Whitney test; n = 10 mice per group) (Table S3b and Fig. S3B). As well, no significant differences are observed in the group of mice with chemogenetic manipulation of AD-projecting RSC neurons (Total locomotor movements: saline, 2236 ± 274.1 cm, n = 10 mice; CNO, 2480 ± 166.2 cm, n = 8 mice; p = 0.408. Percentage of distance traveled in center: saline, 16.9% ± 2.4, n = 10 mice; CNO, 18.4% ± 1.9, n = 8 mice; p = 0.697, Mann-Whitney test) (Table S3b and Fig. S3B). Additionally, the CNO inhibition does not affect average speed in either group (Table S3b). For the M2 group, the average speed was 4.463 ± 0.465 cm/s for Saline and 3.943 ± 0.245 cm/s for CNO (p = 0.4813, Mann-Whitney test), while for the AD group, it was 3.726 ± 0.457 cm/s for saline and 4.134 ± 0.277 cm/s for CNO (p = 0.4082, Mann-Whitney test).

To address potential contributions of RSC in anxiety-related behaviors, we also conducted the elevated plus maze (EPM) test to assess the effects of CNO inhibition of M2-and AD-projecting RSC neurons on mouse behavior. A schematic of the EPM apparatus is shown in Fig. S3C. The results show no significant differences in the number of entries to each arm and the center space between the CNO-treated group and the saline group (n = 10 mice per group, close arm: p = 0.752; center: p = 0.779; open arm: p = 0.561, Mann-Whitney U tests) (Table S3c). Furthermore, the time spent in each location is not affected by the inhibition of M2-projecting RSC neurons (close arm: p = 0.896; center: p = 0.853; open arm: p = 0.896, Mann-Whitney U tests) (Fig. S3D). Similar results are observed in the AD-projecting RSC group where CNO treatment does not affect the entries to nor the time spent in the open arm as compared to control mice treated with saline (# of entries to open arm: p = 0.892; time spent in open arm: p = 0.814, Mann-Whitney U tests, n = 9 mice per group) (Fig. S3D and Table S3c). Therefore, our study suggests that inhibiting RSC neurons projecting to M2 or AD specifically impairs the object location memory without affecting general locomotor activity or anxiety-like behaviors.

**Fig. 6:**
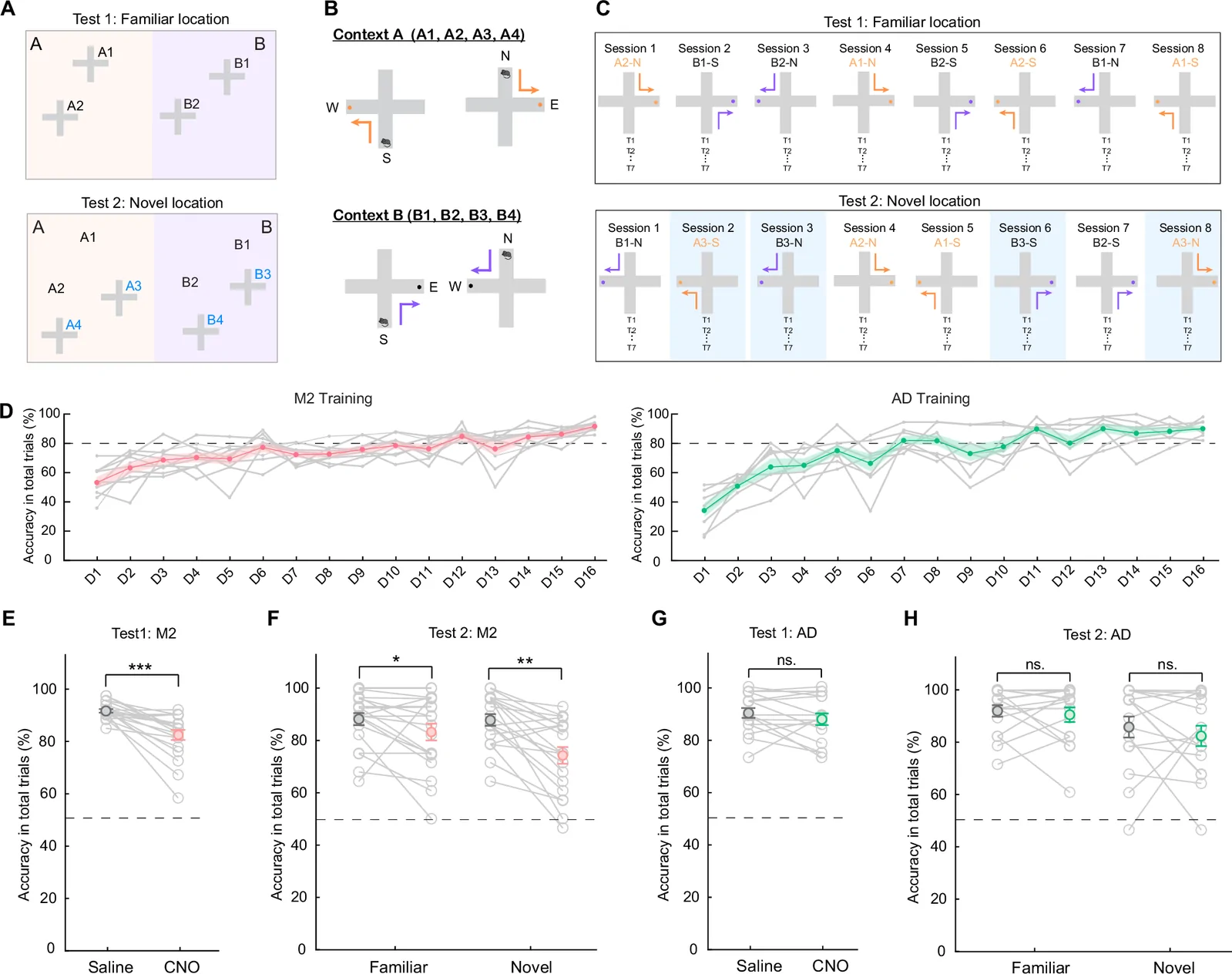
Genetically targeted inactivation of M2-projecting RSC neurons disrupts place-action association in a cross-maze decision-making task. **A** The schematic figure illustrates the place-action test. Top: Overhead view of two visual contexts within the same environment and the maze location layout in Test 1. The experimental room was visually decorated as two compartments, context A (beige side) and context B (light purple side) using distal visual cues and wall coloring. A cross maze, shown in gray and consisting of four identical arms, moves between the two contexts. The four arms were considered as north, south, east, and west relative to the entry door in the room. The main training and testing locations were the four locations labeled as A1 and A2 in context A and B1 and B2 in context B. Bottom: maze location in Test 2. Blue markers indicate additional locations in Test 2 (A3, A4, B3, and B4) for evaluating the transfer of task rules to new locations. **B** Turning rules in each context. In context A, the mouse was trained to make a left turn at all the maze locations, regardless of whether it started from the north or south arms. In context B the mouse was required to make right turns irrespective of start arm and maze location within the context. The starting position of the mouse on each arm of the cross maze is indicated by its location, and the turning direction is indicated by orange and purple arrows. The orange and purple polygons at either east or west arms represent the reward site for context A and B, respectively. **C** Temporal structure of the task. Top: temporal structure of training and Test 1. Each session consisted of seven trials in a particular location with a starting orentation from either the north or south arm before the track was moved to a new location for the next session of seven trials. Each day consisted of 8 sessions, which included all the four  locations and two starting arms (A1-S, A1-N, A2-S, A2-N, B1-S, B1-N, B2-S, B2-N). Location and arm order were randomized and changed every day. Bottom: temporal structure of Test 2. Two novel locations, indicated by the blue shadows, have been added for Test 2. **D** Behavioral accuracy during training. The color-coded dots and lines represent the mean percent accuracy of all the mice across multiple days in each of 4 locations and 2 contexts. Pink represents M2-projecting RSC group (N = 10 mice) while green represents AD-projecting RSC group (N = 8 mice). Mouse performance shows a trend toward higher accuracy over day 1, which gradually increases after a week of training until reaching 80%+ accuracy. Later training improves performance to maintain consistency. The colored shadow indicates the standard error of each group. Each gray line plots the performance of an individual mouse. The dashed line indicates the threshold of 80% accuracy in performance. **E** Performance accuracy of M2 mice in Test 1 is depicted. All mice received CNO or saline 30 min before the first session on each testing day. CNO was administrated via i.p injection at a dose of 5 mg per kg. The accuracy across total trials was calculated by dividing the total number of correct trials by total 56 trials from 8 sessions. All hM4D/CNO mice show significantly impaired accuracy in total trials compared to the control/saline mice. To verify the reliability of CNO results, each test was performed twice, with no differences observed between replicates (Fig. S5). The plot illustrates data from two sets of Test 1. Each light gray line and circle corresponds to an individual mouse. Dark gray represents the mean percent accuracy of control mice with saline treatment, while pink represents hM4D mice with CNO treatment. Data are presented as mean ± s.e.m., and the asterisks, *, ** and *** indicates significant differences as p < 0.05, p < 0.01 and p < 0.001, Wilcoxon signed-rank test. **F** Mean percent accuracy of M2 group in Test 2. The plot illustrates data from two sets of Test 2. Familiar locations included A1, A2, B1 and B2 while novel locations were A3. A4, B3 and B4. CNO inhibition mice exhibit a decrease in the accuracy of total trials in familiar locations, as well as in novel locations compared to saline control mice (**G**, **H**) are arranged in the same way as the panels in (**E**) and (**F**), to show the behavioral performance of the AD group during Tests 1 and 2. The results indicate the CNO inhibition of AD-projecting RSC neurons does not significantly lower the performance accuracy in either familiar or novel locations, compared to the mice in control group.

To examine whether specific RSC circuits facilitate a transformation of spatial information into action, we tested animals’ performance on a novel, cross-maze decision-making task demanding associations of location with choice of appropriate navigational actions. Specifically, the maze task required mice to make left or right turns as determined by the spatial position of the cross maze within a single experimental environment (Fig. 6A). Thus, successful performance in this task required learning a rule-based cognitive partitioning of the allocentric space defined by environmental boundaries [39]. When the maze was placed in context A locations (within the left half of the room), the mouse needed to make a left turn. When it was placed in context B locations (within the right half of the room), the mouse was required to make a right turn. The maze consisted of two starting locations, named north and south, which did not affect the left/right turn task demands (Fig. 6B).

The animals underwent initial training to learn how to make left and right turns at 4 different locations of the cross maze in the environment (2 left half, 2 right half). The maze consisted of an open view structure with walls measuring only 1-2 cm in height, which allowed the mice to see the surrounding distal cues and differentiate environmental locations of the maze. The training began at the north starting point with 12 trials per session and then gradually introduced the south starting point at each location. After a week of training, the sessions were shortened to 7 trials, and additional training was carried out until the animals achieved criterion performance. Each day, the mice underwent a total of 56 trials arranged in 8 sessions of 7 trials with the maze location being moved randomly between the four initial locations (Fig. 6C, top). To assess proficiency in learning the rules, we measured performance by calculating the percentage accuracy across total trials. The training data showed that the performance of all the mice on the task was well above chance (n = 10 mice for M2 group, n = 8 mice for AD group) (Fig. 6D). The accuracy in total trials significantly increased from the first day of training (Fig. 6D). Thus, by the end of the training period the mice had acquired a robust understanding of the appropriate turn choices according to the environmental location of the maze.

To compare the roles of M2-projecting or AD-projecting RSC neurons in transformation of spatial information into action, we used the hM4D/CNO approach to test animals in the four previously trained locations. Every other day, saline or CNO was intraperitoneally injected 30 minutes before the maze task, allowing sufficient time for the metabolization of CNO. The results indicate that with the CNO/hM4D inhibition of M2-projecting RSC neurons, there was a significant reduction in choice accuracy compared to the control saline group (Saline, 92.659% ± 0.70; CNO, 83.21% ± 1.92; p = 0.0003. Wilcoxon signed-rank test, n = 10 mice) (Fig. 6E). The inactivation of the AD-projecting RSC group did not impact performance. With CNO/hM4D inhibition, the mice in the AD group exhibited close to the same level of accuracy as the control group in total trials (Saline, 88.95% ± 2.82; CNO, 86.61% ± 2.98; p > 0.05. Wilcoxon signed-rank test, n = 8 mice) (Fig. 6G). To ensure the robustness of our findings, we repeated Test 1 twice (Fig. S4 and S5A-B). The results confirmed that CNO-induced deficits were consistent across two sets of experiments. The accuracy of mouse performance in the M2 group significantly dropped on both CNO treatment days (D3 and D7) compared to the preceding saline treatment days (D1 and D5) (Saline-D1 vs. CNO-D3, p = 0.0156; Saline-D5 vs. CNO-D7, p = 0.0039; Wilcoxon signed-rank test) (Fig. S5A). To rule out potential effects of CNO on locomotion, we compared the latency of correct trials between CNO and the saline conditions. The results showed no significant differences in the time taken to reach the reward zone (M2 group: Saline, 3.386 ± 0.429 s; CNO, 2.862 ± 0.325 s; p = 0.149; AD group: Saline, 3.788 ± 1.105 s; CNO, 2.214 ± 0.181 s; p = 0.149, Wilcoxon signed-rank test) (Table S3f). For a more comprehensive presentation of the experimental results, we have included detailed behavioral data in Supplementary figures and Tables 3 and 4.

To further investigate the animals’ capability in implementing a rule-based understanding of the environment in a new scenario, we conducted tests for transfer of the left- versus right-turning rule to novel positions within the two sub-regions of the environment (locations A3, A4, B3, B4 of Fig. 6A, bottom). Each testing day, we introduced one novel location, which was randomly mixed with the other four familiar locations throughout the eight sessions (Fig. 6C, bottom). In the novel locations, the saline-treated mice had an accuracy of 88% turning in all trials, whereas inhibiting M2-projecting RSC neurons significantly reduced their performance accuracy to 74% (Saline, 87.86% ± 2.23; CNO, 74.29% ± 3.20; p = 0.0005. Wilcoxon signed-rank test, n = 10 mice) (Fig. 6F). The accuracy in the familiar locations was impaired as well (Saline, 88.21% ± 2.33; CNO, 83.21% ± 3.21; p = 0.0479. Wilcoxon signed-rank test, n = 10 mice per group). No significant differences were found in the AD-projecting RSC group between CNO and saline treatment (Novel locations: saline, 85.93% ± 4.03; CNO, 82.59% ± 3.92; p > 0.05. Wilcoxon signed-rank test, n = 8) (Fig. 6H). Consistent with Test 1, we performed Test 2 twice and found no difference in performance impairment between the two days of CNO treatments (Fig. S5C, D). The accuracy of mouse performance in the M2 group significantly decreased on both CNO treatment days (D3 and D7) following the prior saline treatments on D1 and D5 (Test 2 in novel location: Saline-D1 vs. CNO-D3, p = 0.0215; Saline-D5 vs. CNO-D7, p = 0.0078; Wilcoxon signed-rank test) (Fig. S5C).

The findings indicate that RSC projection-specific subpopulations have distinct behavioral functional roles. Specifically, the M2-projecting RSC neurons are strongly implicated in mediating generalization, to new locations, of previously learned associations between environmental location (or ‘allocentric’ space) with choice of left or right turning actions. Further, our results from both groups show that the rules defining location-action associations can be generalized to new locations.

## Discussion

**Fig. 7:**
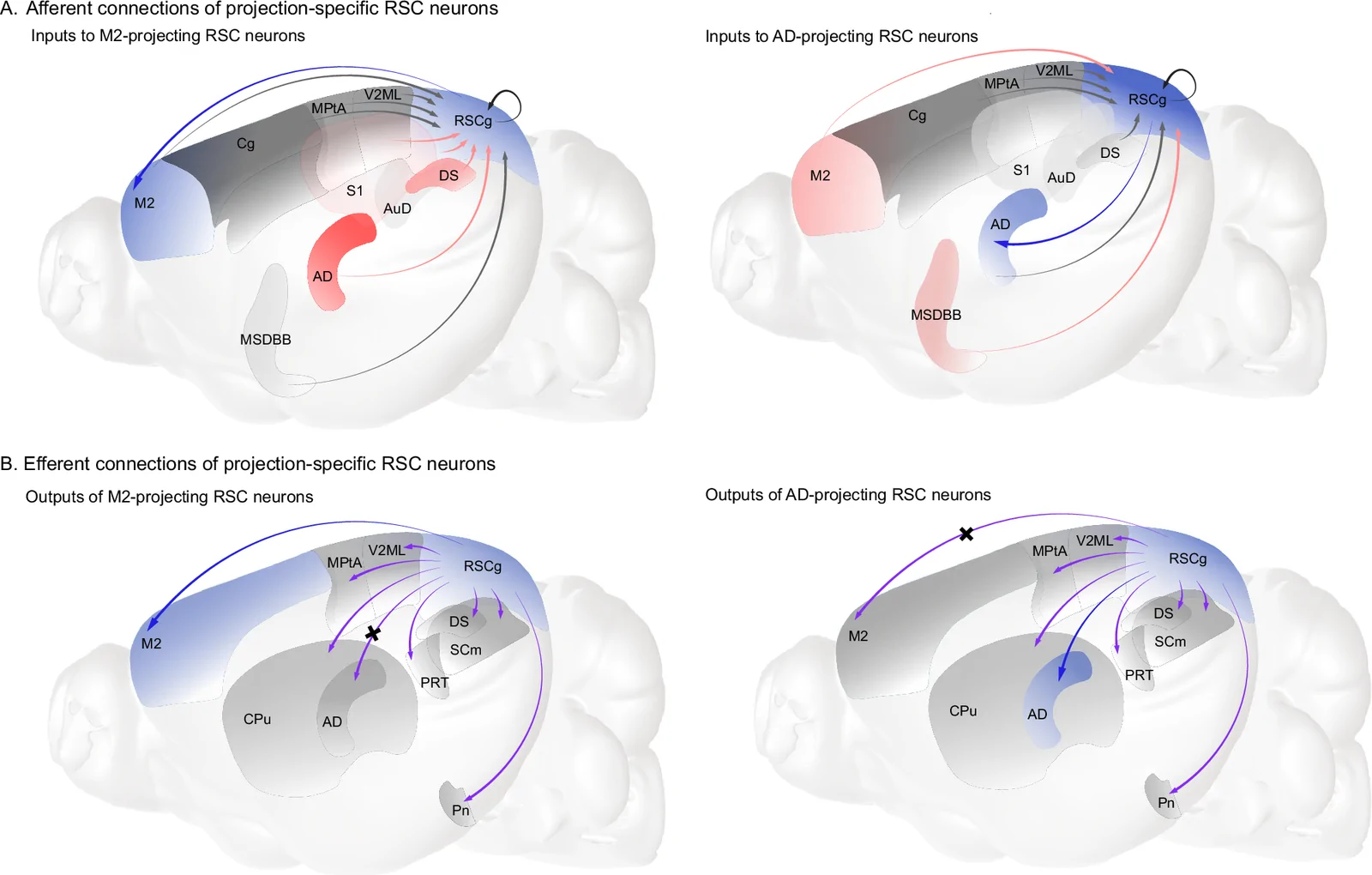
Schematic summary of afferent and efferent connections of M2- or AD-projecting RSCg subpopulations. **A** The summary diagram illustrates the differential inputs to M2- and AD-projecting RSCg neurons. The RSCg to M2 or AD projection is indicated by blue arrow. Pink indicates the mapped input regions that preferentially project to either M2- or AD-projecting RSC neurons. Other major input regions are labeled gray. The gradient colors of pink and gray represent the connectivity strength, with darker shades indicating stronger connections. Input directions are indicated by pink arrows. **B** The diagram follows the same order as in (**A**), to depict the collateral connections of M2- and AD-projecting RSCg neurons. The projection from RSCg to M2 or AD is indicated by blue arrows. Collateral projection regions are indicated by purple arrows. Black ‘X’ indicates no synaptic connection between the two regions.

We identified two subpopulations of RSC neurons defined by their projections to M2 and AD. These populations exhibited a low degree of colocalization within individual neurons and this was consistent with the degree expected by chance. In other words, few AD-projecting RSC neurons also project to M2, and few M2-projecting RSC neurons project to AD. We found minimal evidence for axon terminal fields in AD for M2-projecting RSC neurons, as well as for axon terminal fields in M2 for AD-projecting neurons, which was consistent with the conclusion that M2-projecting and AD-projecting RSC neuron are largely, though not completely, separate populations of neurons. Nevertheless, the same two populations of neurons form overlapping output to other RSC efferent targets such as the pontine nuclei. These RSC sub-populations obtain comparable input from some RSC afferent sources such as posterior parietal cortex. However, AD- and M2-projecting RSC neurons are distinct in that they obtain different distributions of afferents from regions such as subiculum, sensory cortices, septal nucleus, and anterior and laterodorsal thalamus (Fig. 7). The anatomical data thus indicates that these RSC sub-groups integrate different types of information among the diverse set of inputs reaching RSC and can be considered semi-independent circuits both from the perspective of their efferent targets and their afferent sources. Consistent with this conceptualization of RSC’s microcircuitry, circuit-specific inactivation produced dissociable effects on object-location and place-action memory.

A substantial literature has highlighted differences in input sources to intratelencephalic (IT) and corticothalamic (CT) neurons, with IT neurons forming cortico-cortical connections that project to other cortical areas and hemisphere, and CT projecting to the thalamus and postsubiculum [27, 40]. Our study extends this knowledge by focusing on the much more specific cortical and sub-cortical inputs to RSCg neurons having projections to M2 and AD. This focus is complemented by inactivation of a very select group of neurons that significantly impact performance on a subset of multiple behavioral tasks, namely, object location memory and action-place association tasks.

Using multiple synaptic viral tracers, we characterized the connectivity strengths of RSC projection-specific neuron groups and demonstrated that the M2-projecting RSC neurons had stronger connections with the subiculum, anterior and lateral thalamic nuclei, and somatosensory cortex as compared to AD-projecting neurons. Chemogenetic inhibition of M2-projecting neurons impaired rodents’ performance in executing left versus right turns according to the position of a maze intersection in the environment (place-action memory). This strongly suggests that M2-projecting RSC neurons are involved in transforming spatial cognition into action during navigation. Both AD-projecting and M2-projecting subpopulations of RSC neurons played a role in object-location memory despite their sparse colocalization. Altogether, our findings support recent research on the involvement of M2 in converting complex spatial knowledge into action during navigation and provide new insights into the parallel circuitries underlying spatial and mnemonic processing in the RSC [27–29, 41].

The present study used multiple genetically modified viruses to quantitatively compare the strength of connections between two projection-specific subpopulations in the RSC granular area, which distinguishes it from previous research on circuit connectivity [15, 16, 24, 42]. By utilizing a helper AAV and a rabies virus that produce measurable quantities of “starter cells” at the injection site, we can effectively evaluate the strength of input connections (CSI) in a quantitative manner [32, 37, 43]. By combining the rabies virus with the rAAV2-retro-cre virus, we were able to precisely target subregion-, sublayer-, and projection-specific neurons in the deep layer of granular RSC. This enabled us to examine the quantitative CSIs for the brain-wide and diverse sources of afferents innervating projection-specific RSC subpopulations. The tracing results reveal that the subiculum, anterior dorsal and lateral dorsal thalamic nuclei, and sensory cortices provide stronger innervation to M2-projecting RSCg neurons located at granular layer 5 as compared to AD-projecting RSCg neurons. Furthermore, use of synaptoTAG2, a synaptic terminal targeting virus [31], enabled a quantitative comparison of collateral projections from these projection-specific RSC neurons, providing further insight into the composition of RSC circuits and their potential behavioral functions. While our study focused on whether RSCg, RSCd neurons may exhibit different afferent sources. Exploring these differences between projection-specific neurons in RSCd and RSCg represents a valuable direction for future research.

Previous studies have anatomically delineated RSC into distinct regions [13–15] and identified intrinsic connections among these subregions [42]. These findings suggest that various RSC subregions may serve different yet complementary roles in spatial learning and memory functions [44–48]. However, quantitative assessments of local inputs are lacking, resulting in uncertainty regarding the presence of distinct intrinsic connections and their importance in supporting interaction between RSC sub-regions and their roles in cognition. In our study, we addressed this gap by quantitatively measuring the strength of local inputs from all RSC subregions (RSCd, RSCg-a, RSCg-b, and RSC-c) to projection-specific RSC sub-populations along the rostro-caudal (longitudinal) axis. Our findings confirm that RSC subregions interact with each other, with the strongest local inputs to RSCg-c originating from the RSCg-c itself, but with appreciable input from dysgranular cortex onto both types of projection-specific neurons. Lesion studies have indicated RSCg-a/b and RSCg-c function independently in spatial learning and memory [45, 47], as well as across the anterior/posterior axis [49]. This distinct pattern was further supported by our discovery that in comparison with the strong inputs from RSCg-c, RSCg-a and b sparsely project to the RSCg-c region. Consistent with behavioral differences across the rostro-caudal axis [49], our quantification data clearly provide evidence of variations in intrinsic connection strength along the anterior/posterior axis, with stronger inputs observed from rostral to caudal RSC regions and weaker inputs in the reverse direction. A somewhat unexpected finding is that intrinsic connections are very strong as compared to extrinsic input regions and stronger for AD-projecting than M2-projecting RSC neurons. The functional implications of intrinsic connectivity patterns across RSC sub-regions on behavior and neural dynamics remain to be determined in future work, but indicate that dysgranular RSC and RSCg-c may exhibit separate function from RSCg-a/b.

While the functional roles of RSC have been extensively investigated in both rodents and humans, existing rodent behavioral studies have often utilized broad lesions encompassing all RSC subregions, spanning from the rostral to caudal regions [5, 38, 47, 49, 50]. Recent studies provided compelling evidence that subsets of projection-specific neurons within the subiculum and hippocampus can play crucial functional roles in object/place learning even when they represent small fractions of the total number of neurons [34, 51]. Furthermore, anatomical and physiological studies suggest RSC should be considered in a subregion-specific [26], layer-specific [22, 52], pathway-specific [27], and cell-type-specific manner [52]. In the present study, we used the DREADD system to selectively inhibit RSC subpopulations, offering a more circuit-based approach compared to traditional lesion studies. By using chemogenetic inhibition of RSC sub-populations during performance of spatial memory tasks, we were able to compare the cognitive and behavioral functions of M2- and AD-projecting RSC neurons. Our findings, consistent with previous lesion studies impacting much larger populations of RSC neurons [38], showed that inhibiting both projection-specific sub-populations of RSC neurons disrupted object-location memory but not object-recognition memory.

Given the preponderance of head-direction sensitive neurons within AD [50], one might interpret the effects of inhibition of AD-projecting RSC neurons on object-location memory as indicating that this sub-population plays a role in encoding memories for orientation relationships between environmental boundaries and the arrangement of environmental landmarks (objects) within those boundaries. Additionally, the cholinergic projections from the medial septum and the horizontal and vertical limb of diagonal band might contribute to cue-specific learning and memory [53–56].

Given the preponderance of neurons tuned to navigational actions in M2 [41], one might interpret the impact of inhibiting M2-projecting RSC neurons on OLM as an indication that these neurons are responsible for the generation of exploratory actions in response to alterations in the spatial relationships between landmarks (objects) and environmental boundaries. The results of the present work cannot directly support such conclusions but can inform the design of future studies that can address highly specific functions of RSC neuron sub-populations.

Recent studies have considered multiple pathways through RSC that may contribute to different behavioral functions in spatial memory. These pathways may include multiple complementary routes, such as from hippocampus to anterodorsal thalamus via the fornix or another from hippocampus to RSC to AD, to mediate spatial working memory [57]. Alternatively, a parallel pathway may innervate RSC neurons through collateral connections from the subiculum and other upstream regions to regulate spatial or emotional memory [22, 26]. Our behavioral data supports the hypotheses that different subpopulations of RSC neurons may be involved in distinct behavioral functions or support similar functions through semi-independent pathways. Both M2- and AD-projecting neurons facilitate “where” memory during the object-location spatial memory test, but neither in “what” memory for recognizing a novel object nor in stress-related behaviors. In the present work, M2-projecting RSC neurons, but not AD-projecting RSC neurons, were found to impact the association of environmental locations with specific navigational actions as revealed by the place-action task. In this task, associations of locations with specific actions are demanded, but head orientation is irrelevant to task performance. Thus, the result is consistent with both the preponderance of navigational action-related neurons in M2 [41] and data from the present work demonstrating significantly stronger input to M2-projecting RSC neurons from brain regions such as subiculum that encode environmental locations.

Neurophysiological data from Olson et al. [41] strongly indicated that M2 is crucial for transforming complex spatial cognition into action. Manipulations of the RSC to M2 connection was also implicated in utilization of visual cues to guide action selection at a virtual maze intersection [28]. Our tracing and behavioral data provide convincing evidence that M2-projecting RSC neurons play a key role in transforming spatial information from subiculum and orientation information from anterior and lateral dorsal thalamic nuclei into action tuning in M2 to guide navigation-related action-taking. Because the distribution of these afferents to RSC sub-groups differs, it is possible that these inputs are key to directly regulating the downstream responses to RSC in M2. Although M2-projecting and AD-projecting neurons receive equivalent strength input from other brain regions with neural activity related to location, orientation, and landmarks, such as the parietal cortex [58–60], postsubiculum [61], and visual cortex [48, 62], the inhibition of M2-projecting, but not AD-projecting RSC neurons, impairs performance on the action-location memory task. The impairments in OLM, but not ORM, following inhibition of AD-projecting RSC neurons indicates that AD-projecting RSC neurons integrate information related to the location of objects/landmarks in the environment or their orientation relative to boundaries. This same information might be related to updating of orientation or other functions outside of space-to-action transformations [50]. These findings imply that the transformation of spatial information from the subiculum and thalamic nuclei into navigational actions involves M2-projecting RSC neurons rather than the RSC neurons projecting to AD. Given the less than complete effect of our manipulations on task performance, our behavioral findings support the notion that connecting place to action may involve multiple circuits in the brain [63–65]. Although performance accuracy in the M2 group was notably impaired in the place-action task, hovering around 80%, it underscores the role of alternate pathways in supporting working memory and complex tasks like place-action, which necessitate actions, orientation, and other cognitive functions.

The distribution of intrinsic RSC connections for AD- versus M2-projecting RSC neurons provides insight into how sub-populations of RSC neurons integrate local feedback information and how that may relate to spatial memory [66, 67]. A successful navigation system requires that the network be able to consistently update surrounding spatial information to match movements. Theoretically, this can be achieved through feedforward and feedback connections via reciprocal connections with multiple brain regions or via the intrinsic connections within the subpopulation of RSC pathways carrying different forms of spatial information. Our findings suggest that the strong intrinsic connections within RSC subregions allow individual retrosplenial regions to process distinct data in a relatively autonomous manner while also utilizing the intrinsic connections to other subpopulations and subregions for integrating information [67]. In cases where the mouse turns into a wrong arm without reward during the first trial of a new session, other projection-specific pathways that regulate reward, head direction, or visual stimulus can immediately exchange information with M2-projecting RSC subpopulations to correct the turning action in the next trial, which may also contribute to the planning process.

### Limitations of the study

One limitation of the present study is the possibility that the inhibition of CNO may have also affected collateral connections, as the AAV-synaptoTAG2 data demonstrate that both AD-projecting and M2-projecting neurons overlap in their projections to brain regions other than AD and M2. Both M2-projecting and AD-projecting RSC neurons innervate multiple output regions, most of which are related to motion and orienting movements [68–70]. While the open field exploration results exclude the influence of CNO on motor activity more generally, it is possible that RSC outputs to regions other than M2 may also be involved in coding action-location associations. To address the potential CNO issue, future experiments utilizing the optogenetics system to selectively inhibit or activate RSC synaptic terminals that directly innervate the M2 region. This approach may offer a better means to confirm the function of the specific RSC projections crucial for place-action and OLM performance [71], conducting optogenetic manipulations of synaptic terminals may be better suited to eliminating concerns regarding the potential off-target effect of CNO.

In conclusion, our work supports the idea that the M2-projecting RSC pathway is involved in the processing and segregation of upstream spatial information, which is then transmitted to the downstream motor cortex to regulate actions and decision-making. These findings also shed new light on the composition of neural circuits in RSC, including their specific roles in spatial memory and behavioral control and the extent to which RSC can be conceptualized as home to a variety of semi-independent circuits with differential contributions to cognition and behavior.

## Supplementary information

Supplementary figure legend

Supplementary figure 1

Supplementary figure 2

Supplementary figure 3

Supplementary figure 4

Supplementary figure 5

Supplementary table 1: Viral information

Supplementary table 2: Quantitative Analysis and Statistical Summary of Viral Tracing Experiment

Supplementary table 3:Quantitative Analysis and Statistical Summary of Behavioral Experiments

Supplementary Table 4: raw data

## Data Availability

All data generated or analysed during this study are included in this published article and its supplementary information files.

## References

[1] Vann SD, Aggleton JP, Maguire EA. What does the retrosplenial cortex do? Nat Rev Neurosci. 2009;10:792–802. 19812579 10.1038/nrn2733

[2] Maguire EA. The retrosplenial contribution to human navigation: a review of lesion and neuroimaging findings. Scand J Psychol. 2001;42:225–38. 11501737 10.1111/1467-9450.00233

[3] Miller AMP, Mau W, Smith DM. Retrosplenial cortical representations of space and future goal locations develop with learning. Curr Biol. 2019;29:2083–2090.e2084. 31178316 10.1016/j.cub.2019.05.034 PMC6637961

[4] Cooper BG, Mizumori SJ. Retrosplenial cortex inactivation selectively impairs navigation in darkness. Neuroreport. 1999;10:625–30. 10208601 10.1097/00001756-199902250-00033

[5] Cooper BG, Manka TF, Mizumori SJ. Finding your way in the dark: the retrosplenial cortex contributes to spatial memory and navigation without visual cues. Behav Neurosci. 2001;115:1012–28. 11584914 10.1037//0735-7044.115.5.1012

[6] Bluhm RL, Miller J, Lanius RA, Osuch EA, Boksman K, Neufeld RW, et al. Retrosplenial cortex connectivity in schizophrenia. Psychiatry Res. 2009;174:17–23. 19783410 10.1016/j.pscychresns.2009.03.010

[7] Klimczak P, Rizzo A, Castillo-Gomez E, Perez-Rando M, Gramuntell Y, Beltran M, et al. Parvalbumin interneurons and perineuronal nets in the hippocampus and retrosplenial cortex of adult male mice after early social isolation stress and perinatal NMDA receptor antagonist treatment. Front synaptic Neurosci. 2021;13:733989. 34630066 10.3389/fnsyn.2021.733989 PMC8493248

[8] Corcoran KA, Yamawaki N, Leaderbrand K, Radulovic J. Role of retrosplenial cortex in processing stress-related context memories. Behav Neurosci. 2018;132:388–95. 29878804 10.1037/bne0000223 PMC6188831

[9] Vogt BA, Peters A. Form and distribution of neurons in rat cingulate cortex: areas 32, 24, and 29. J Comp Neurol. 1981;195:603–25. 7462444 10.1002/cne.901950406

[10] Vogt BA, Paxinos G. Cytoarchitecture of mouse and rat cingulate cortex with human homologies. Brain Struct Funct. 2014;219:185–92. 23229151 10.1007/s00429-012-0493-3

[11] Aggleton JP, Yanakieva S, Sengpiel F, Nelson AJ. The separate and combined properties of the granular (area 29) and dysgranular (area 30) retrosplenial cortex. Neurobiol Learn Mem. 2021;185:107516. 34481970 10.1016/j.nlm.2021.107516

[12] Sugar J, Witter MP, van Strien NM, Cappaert NL. The retrosplenial cortex: intrinsic connectivity and connections with the (para)hippocampal region in the rat. An interactive connectome. Front Neuroinform. 2011;5:7. 21847380 10.3389/fninf.2011.00007 PMC3147162

[13] van Groen T, Wyss JM. Connections of the retrosplenial dysgranular cortex in the rat. J Comp Neurol. 1992;315:200–16. 1545009 10.1002/cne.903150207

[14] van Groen T, Wyss JM. Connections of the retrosplenial granular a cortex in the rat. J Comp Neurol. 1990;300:593–606. 2273095 10.1002/cne.903000412

[15] Van Groen T, Wyss JM. Connections of the retrosplenial granular b cortex in the rat. J Comp Neurol. 2003;463:249–63. 12820159 10.1002/cne.10757

[16] Vogt BA, Miller MW. Cortical connections between rat cingulate cortex and visual, motor, and postsubicular cortices. J Comp Neurol. 1983;216:192–210. 6863602 10.1002/cne.902160207

[17] Aggleton JP, Wright NF, Vann SD, Saunders RC. Medial temporal lobe projections to the retrosplenial cortex of the macaque monkey. Hippocampus. 2012;22:1883–1900. 22522494 10.1002/hipo.22024 PMC3510309

[18] Witter MP, Ostendorf RH, Groenewegen HJ. Heterogeneity in the dorsal subiculum of the rat. distinct neuronal zones project to different cortical and subcortical targets. Eur J Neurosci. 1990;2:718–25. 12106290 10.1111/j.1460-9568.1990.tb00462.x

[19] Burwell RD, Amaral DG. Cortical afferents of the perirhinal, postrhinal, and entorhinal cortices of the rat. J Comp Neurol. 1998;398:179–205. 9700566 10.1002/(sici)1096-9861(19980824)398:2<179::aid-cne3>3.0.co;2-y

[20] Jones BF, Witter MP. Cingulate cortex projections to the parahippocampal region and hippocampal formation in the rat. Hippocampus. 2007;17:957–76. 17598159 10.1002/hipo.20330

[21] Kerr KM, Agster KL, Furtak SC, Burwell RD. Functional neuroanatomy of the parahippocampal region: the lateral and medial entorhinal areas. Hippocampus. 2007;17:697–708. 17607757 10.1002/hipo.20315

[22] Brennan EK, Jedrasiak-Cape I, Kailasa S, Rice SP, Sudhakar SK, Ahmed OJ. Thalamus and claustrum control parallel layer 1 circuits in retrosplenial cortex. *eLife* 2021; 10. 10.7554/eLife.62207 PMC8233040 34170817

[23] Sripanidkulchai K, Wyss JM. Thalamic projections to retrosplenial cortex in the rat. J Comp Neurol. 1986;254:143–65. 3794004 10.1002/cne.902540202

[24] Shibata H. Organization of projections of rat retrosplenial cortex to the anterior thalamic nuclei. Eur J Neurosci. 1998;10:3210–9. 9786214 10.1046/j.1460-9568.1998.00328.x

[25] Kamishina H, Conte WL, Patel SS, Tai RJ, Corwin JV, Reep RL. Cortical connections of the rat lateral posterior thalamic nucleus. Brain Res. 2009;1264:39–56. 19368845 10.1016/j.brainres.2009.01.024

[26] Lomi E, Mathiasen ML, Cheng HY, Zhang N, Aggleton JP, Mitchell AS, et al. Evidence for two distinct thalamocortical circuits in retrosplenial cortex. Neurobiol Learn Mem. 2021;185:107525. 34555510 10.1016/j.nlm.2021.107525

[27] Yamawaki N, Radulovic J, Shepherd GM. A corticocortical circuit directly links retrosplenial cortex to M2 in the mouse. J Neurosci : Off J Soc Neurosci. 2016;36:9365–74. 10.1523/JNEUROSCI.1099-16.2016 PMC5013186 27605612

[28] Franco LM, Goard MJ. A distributed circuit for associating environmental context with motor choice in retrosplenial cortex. *Sci Adv.* 2021; 7. 10.1126/sciadv.abf9815 PMC8386923 34433557

[29] Alexander AS, Nitz DA. Retrosplenial cortex maps the conjunction of internal and external spaces. Nat Neurosci. 2015;18:1143–51. 26147532 10.1038/nn.4058

[30] Tervo DG, Hwang BY, Viswanathan S, Gaj T, Lavzin M, Ritola KD, et al. A designer AAV variant permits efficient retrograde access to projection neurons. Neuron. 2016;92:372–82. 27720486 10.1016/j.neuron.2016.09.021 PMC5872824

[31] Xu W, Sudhof TC. A neural circuit for memory specificity and generalization. Science. 2013;339:1290–5. 23493706 10.1126/science.1229534 PMC3651700

[32] Wickersham IR, Lyon DC, Barnard RJ, Mori T, Finke S, Conzelmann KK, et al. Monosynaptic restriction of transsynaptic tracing from single, genetically targeted neurons. Neuron. 2007;53:639–47. 17329205 10.1016/j.neuron.2007.01.033 PMC2629495

[33] Vogel-Ciernia A, Wood MA. Examining object location and object recognition memory in mice. Curr Protoc Neurosci. 2014;69:8 31 31–17. 10.1002/0471142301.ns0831s69 PMC4219523 25297693

[34] Lin X, Amalraj M, Blanton C, Avila B, Holmes TC, Nitz DA, et al. Noncanonical projections to the hippocampal CA3 regulate spatial learning and memory by modulating the feedforward hippocampal trisynaptic pathway. PLoS Biol. 2021;19:e3001127. 34928938 10.1371/journal.pbio.3001127 PMC8741299

[35] Paxinos G, Franklin KBJ. *The mouse brain in stereotaxic coordinates*. Compact 2nd edn. Elsevier Academic Press: Amsterdam ; Boston, 2004.

[36] Sun Y, Nguyen AQ, Nguyen JP, Le L, Saur D, Choi J, et al. Cell-type-specific circuit connectivity of hippocampal CA1 revealed through Cre-dependent rabies tracing. Cell Rep. 2014;7:269–80. 24656815 10.1016/j.celrep.2014.02.030 PMC3998524

[37] Wall NR, Wickersham IR, Cetin A, De La Parra M, Callaway EM. Monosynaptic circuit tracing in vivo through Cre-dependent targeting and complementation of modified rabies virus. Proc Natl Acad Sci USA. 2010;107:21848–53. 21115815 10.1073/pnas.1011756107 PMC3003023

[38] Vann SD, Aggleton JP. Extensive cytotoxic lesions of the rat retrosplenial cortex reveal consistent deficits on tasks that tax allocentric spatial memory. Behav Neurosci. 2002;116:85–94. 11895186

[39] Shelley LE, Barr CI, Nitz DA. Cortical and hippocampal dynamics under logical fragmentation of environmental space. Neurobiol Learn Mem. 2022;189:107597. 35134554 10.1016/j.nlm.2022.107597

[40] Harris KD, Shepherd GM. The neocortical circuit: themes and variations. Nat Neurosci. 2015;18:170–81. 25622573 10.1038/nn.3917 PMC4889215

[41] Olson JM, Li JK, Montgomery SE, Nitz DA. Secondary motor cortex transforms spatial information into planned action during navigation. Curr Biol. 2020;30:1845–1854.e1844. 32302586 10.1016/j.cub.2020.03.016

[42] Shibata H, Honda Y, Sasaki H, Naito J. Organization of intrinsic connections of the retrosplenial cortex in the rat. Anat Sci Int. 2009;84:280–92. 19322631 10.1007/s12565-009-0035-0

[43] Luo L, Callaway EM, Svoboda K. Genetic dissection of neural circuits: a decade of progress. Neuron. 2018;98:256–81. 29673479 10.1016/j.neuron.2018.03.040 PMC5912347

[44] Pothuizen HH, Davies M, Aggleton JP, Vann SD. Effects of selective granular retrosplenial cortex lesions on spatial working memory in rats. Behav Brain Res. 2010;208:566–75. 20074589 10.1016/j.bbr.2010.01.001

[45] Pothuizen HH, Davies M, Albasser MM, Aggleton JP, Vann SD. Granular and dysgranular retrosplenial cortices provide qualitatively different contributions to spatial working memory: evidence from immediate-early gene imaging in rats. Eur J Neurosci. 2009;30:877–88. 19712100 10.1111/j.1460-9568.2009.06881.x

[46] Vann SD, Aggleton JP. Selective dysgranular retrosplenial cortex lesions in rats disrupt allocentric performance of the radial-arm maze task. Behav Neurosci. 2005;119:1682–6. 16420172 10.1037/0735-7044.119.6.1682

[47] van Groen T, Kadish I, Wyss JM. Retrosplenial cortex lesions of area Rgb (but not of area Rga) impair spatial learning and memory in the rat. Behav Brain Res. 2004;154:483–91. 15313037 10.1016/j.bbr.2004.03.016

[48] Hindley EL, Nelson AJ, Aggleton JP, Vann SD. The rat retrosplenial cortex is required when visual cues are used flexibly to determine location. Behav Brain Res. 2014;263:98–107. 24486256 10.1016/j.bbr.2014.01.028 PMC3969719

[49] Sutherland RJ, Whishaw IQ, Kolb B. Contributions of cingulate cortex to two forms of spatial learning and memory. J Neurosci : Off J Soc Neurosci. 1988;8:1863–72. 10.1523/JNEUROSCI.08-06-01863.1988 PMC6569344 3385478

[50] Clark BJ, Bassett JP, Wang SS, Taube JS. Impaired head direction cell representation in the anterodorsal thalamus after lesions of the retrosplenial cortex. J Neurosci : Off J Soc Neurosci. 2010;30:5289–302. 10.1523/JNEUROSCI.3380-09.2010 PMC2861549 20392951

[51] Sun Y, Jin S, Lin X, Chen L, Qiao X, Jiang L, et al. CA1-projecting subiculum neurons facilitate object-place learning. Nat Neurosci. 2019;22:1857–70. 31548723 10.1038/s41593-019-0496-y PMC6819262

[52] Kinnavane L, Vann SD, Nelson AJD, O’Mara SM, Aggleton JP. Collateral Projections Innervate the Mammillary Bodies and Retrosplenial Cortex: A New Category of Hippocampal Cells. *eNeuro* 2018; **5**. 10.1523/ENEURO.0383-17.2018 PMC5844061 29527569

[53] Stewart DJ, MacFabe DF, Leung LW. Topographical projection of cholinergic neurons in the basal forebrain to the cingulate cortex in the rat. Brain Res. 1985;358:404–7. 4075131 10.1016/0006-8993(85)90994-1

[54] Baxter MG, Holland PC, Gallagher M. Disruption of decrements in conditioned stimulus processing by selective removal of hippocampal cholinergic input. J Neurosci : Off J Soc Neurosci. 1997;17:5230–6. 10.1523/JNEUROSCI.17-13-05230.1997 PMC6573295 9185560

[55] Tengelsen LA, Robertson RT, Yu J. Basal forebrain and anterior thalamic contributions to acetylcholinesterase activity in granular retrosplenial cortex of rats. Brain Res. 1992;594:10–18. 1467929 10.1016/0006-8993(92)91024-9

[56] Todd TP, Fournier DI, Bucci DJ. Retrosplenial cortex and its role in cue-specific learning and memory. Neurosci Biobehav Rev. 2019;107:713–28. 31055014 10.1016/j.neubiorev.2019.04.016 PMC6906080

[57] Dumont JR, Petrides M, Sziklas V. Fornix and retrosplenial contribution to a hippocampo-thalamic circuit underlying conditional learning. Behav Brain Res. 2010;209:13–20. 20060426 10.1016/j.bbr.2009.12.040

[58] Olsen GM, Ohara S, Iijima T, Witter MP. Parahippocampal and retrosplenial connections of rat posterior parietal cortex. Hippocampus. 2017;27:335–58. 28032674 10.1002/hipo.22701

[59] Nitz D. Parietal cortex, navigation, and the construction of arbitrary reference frames for spatial information. Neurobiol Learn Mem. 2009;91:179–85. 18804545 10.1016/j.nlm.2008.08.007

[60] Nitz DA. Tracking route progression in the posterior parietal cortex. Neuron. 2006;49:747–56. 16504949 10.1016/j.neuron.2006.01.037

[61] Lozano YR, Page H, Jacob PY, Lomi E, Street J, Jeffery K. Retrosplenial and postsubicular head direction cells compared during visual landmark discrimination. Brain Neurosci Adv. 2017;1:2398212817721859. 30246155 10.1177/2398212817721859 PMC6124005

[62] Niell CM, Stryker MP. Modulation of visual responses by behavioral state in mouse visual cortex. Neuron. 2010;65:472–9. 20188652 10.1016/j.neuron.2010.01.033 PMC3184003

[63] Jadhav SP, Rothschild G, Roumis DK, Frank LM. Coordinated excitation and inhibition of prefrontal ensembles during awake hippocampal sharp-wave ripple events. Neuron. 2016;90:113–27. 26971950 10.1016/j.neuron.2016.02.010 PMC4824654

[64] McLaughlin AE, Diehl GW, Redish AD. Potential roles of the rodent medial prefrontal cortex in conflict resolution between multiple decision-making systems. Int Rev Neurobiol. 2021;158:249–81. 33785147 10.1016/bs.irn.2020.11.009 PMC8211383

[65] Papale AE, Zielinski MC, Frank LM, Jadhav SP, Redish AD. Interplay between hippocampal sharp-wave-ripple events and vicarious trial and error behaviors in decision making. Neuron. 2016;92:975–82. 27866796 10.1016/j.neuron.2016.10.028 PMC5145752

[66] Cho J, Sharp PE. Head direction, place, and movement correlates for cells in the rat retrosplenial cortex. Behav Neurosci. 2001;115:3–25. 11256450 10.1037/0735-7044.115.1.3

[67] Miller AMP, Serrichio AC, Smith DM. Dual-factor representation of the environmental context in the retrosplenial cortex. Cereb Cortex. 2021;31:2720–8. 33386396 10.1093/cercor/bhaa386 PMC8023839

[68] Wagner MJ, Kim TH, Kadmon J, Nguyen ND, Ganguli S, Schnitzer MJ, et al. Shared cortex-cerebellum dynamics in the execution and learning of a motor task. Cell. 2019;177:669–682 e624. 30929904 10.1016/j.cell.2019.02.019 PMC6500577

[69] Li YT, Turan Z, Meister M. Functional architecture of motion direction in the mouse superior colliculus. Curr Biol. 2020;30:3304–3315.e3304. 32649907 10.1016/j.cub.2020.06.023 PMC8221388

[70] Masullo L, Mariotti L, Alexandre N, Freire-Pritchett P, Boulanger J, Tripodi M. Genetically defined functional modules for spatial orienting in the mouse superior colliculus. Curr Biol. 2019;29:2892–2904.e2898. 31474533 10.1016/j.cub.2019.07.083 PMC6739420

[71] Lopez AJ, Kramar E, Matheos DP, White AO, Kwapis J, Vogel-Ciernia A, et al. Promoter-specific effects of DREADD modulation on hippocampal synaptic plasticity and memory formation. J Neurosci : Off J Soc Neurosci. 2016;36:3588–99. 10.1523/JNEUROSCI.3682-15.2016 PMC4804014 27013687

