# Supplementary material for: Projection-specific circuits of retrosplenial cortex with differential contributions to spatial cognition
Source: Mol Psychiatry. 2024 Nov 7;30(5):2068–84. doi: 10.1038/s41380-024-02819-8 (PMC12014379; doi:10.1038/s41380-024-02819-8)
Supplement: Supplementary file 1 — Supplementary figure legend [file 41380_2024_2819_MOESM1_ESM.docx]

**Supplementary legends**

**Figure S1. Cre-dependent AAV synaptoTAG2 virus maps the projections of M2- and AD-projecting RSCg neurons.**

**(A)** Representative immunostaining images showing Cre-positive neurons at M2 injection site. Brain sections labeled with rAAV2-retro-Cre were stained with a Cy5-conjugated secondary antibody, visualized as pink in the magnified panel on the right. **(B)** Coronal sections of the RSCg injection regions of anterograde tracing of synaptic connections of M2-projecting RSCg neurons. Injection sites are shown in white boxes with magnified images on the right four panels. The tdTomato labels the whole cell while the GFP labels expression in synaptic terminals. DAPI is shown in blue. (**C-D**) follow the same order as **A-B** and reveal the Cre staining and the RSCg injection site of AD-projecting RSCg group. (**E-I**) Mapping of the projection regions of AD-projecting RSCg neurons. The panels are arranged by low (10x microscopy, left panels) and high-resolution 20 X confocal images (right panels). The red tdTomato labeling marks axonal fibers, while green GFP-labeling marks synapses projecting from the AD-projecting neurons. Yellow overlap of red and green labeling indicates the presence of synaptic connections. The projections brain regions include the caudate putamen (CPu), parietal cortex, medial (MPtA) and lateral (LPtA), anterior cingulate cortex (ACC), postsubiculum (PostSub), secondary visual cortex, medial and lateral subregions (V2ML and V2L), Pontine nuclei (Pn) and superior coliculus (DpG). (**J**) Quantitative analysis of fluorescence intensity in the M2-projecting (left panel) and AD-projecting (right panel) RSCg output regions. Each data point represents the mean fluorescence intensity from 3 brain sections per region. N = 2 mice per group. (**K**) Percentage of neurons labeled in the M2 and AD injection sites, along with corresponding fluorescence-labeled RSC input neurons in retrograde AAV2-mRuby and GFP tracing experiment. Each circle represents the mean of fluorescence-labeled neurons from 4 brain sections. N = 4 mice per group.

Figure S2. Cre-dependent monosynaptic rabies tracing reveals local and distant circuit input connections to M2- and AD- projecting RSCg subpopulations.

**(A).** Quantitative measurements of input connection strengths by the CSI following rabies tracing from M2- and AD-projecting RSCg neurons. The data were measured from 10 C57 mice with n = 5 mice per RSCg subpopulation. All data are presented as mean ± SE. The significance of differences for each input region was tested using the Mann-Whitney U test, followed by the False Discovery Rate (FDR) multiple comparison test, with statistical significance set at *P < 0.05. **(B)**. Cingulate and local RSC inputs along the anterior-posterior axis. The cingulate cortex is divided into anterior cingulate cortex (ACC) and medial cingulate cortex (MCC) according to the distance from the bregma. The RSCg injection area is highlighted by gray shadow. Abbreviations: ACC1/2: anterior cingulate cortex, area 1 or 2; AD, anterodorsal thalamic nucleus; AM, anteromedial thalamic nucleus; AuD, secondary auditory cortex; AV, anteroventral thalamic nucleus; DS, dorsal subiculum; LD, laterodorsal thalamic nucleus; LP, lateral posterior thalamic nucleus; LPtA, lateral parietal association cortex; M1, primary motor cortex; M2, secondary motor cortex; MCC1/2: medium cingulate cortex, area 1 or 2; MPtA, medial parietal association cortex; MSDBB, medial septum and diagonal band of Broca (including horizontal and vertical diagonal band); PostSub, post subiculum; RSCd, retroslenial cortex, dysgrannular area; RSCg, retrosplenial cortex, granular area; S1, primary somatosensory cortex; V1, primary visual cortex; V2ML, secondary visual cortex, mediolateral region; V2MM, secondary visual cortex, mediomedial region.

**Figure S3. Genetically targeted inactivation of M2-projecting or AD-projecting RSCg neurons do not affect locomotor activities and anxiety.**

(**A**) Illustration of the open field test. The locomotion test chamber measures 40 cm by 40 cm. The center zone, depicted by a blue shadow, is 25.8 cm by 25.8 cm. CNO (5mg per kg, ip) or saline was administered to experimental mice 30 minutes before testing. (**B**) Open field results following CNO/hM4D- inactivation of M2- and AD-projecting RSCg neurons. The percentage of distance traveled by mice in the center zone relative to the total distance traveled in the chamber in 10 min was measured. The left and right panels show the impact of CNO inactivation on M2 and AD-projecting RSCg neurons, respectively. No significant differences in locomotor activities were observed when compared to control saline groups (M2, n= 10 mice, p = 0.971; AD, n = 9 mice, p = 0.175; Mann-Whitney *U*-test). Each data point is represented as a color-coded circle, with black indicating the saline treated control group, pink indicating the CNO treated M2-projecting RSCg group, and green indicating CNO treated AD-projecting RSCg group. The data is presented as the mean ± s.e.m., and nonsignificant differences are denoted by ns. (**C**) Schematic illustration of the elevated plus maze (EPM) test. The EPM apparatus comprises two open and two closed arms, each 25 cm × 5 cm measuring, with a height of 50 cm from the ground. The mice were initially placed in the center zone and allowed to explore for 3 min. All of the mice received a dose of CNO (5mg per kg, ip) or saline 30 minutes before the test. (**D**) EPM results following CNO/hM4D-inactivation of M2- and AD-projecting RSCg neurons. The time spent in each location (closed arms, center, and open arms) were measured and no significant differences were observed between the control saline and CNO-treated groups (n = 10 mice for M2, n = 9 mice for AD, Mann-Whitney *U*-test). (**E-F**) Representative immunostaining images showing Cre-positive neurons at M2 and AD injection sites, respectively. Brain sections labeled with rAAV2-retro-Cre were stained with a Cre- primary antibody, followed with a Cy5-conjugated secondary antibody, visualized as pink in the magnified panel on the right. The scale bar (500 μm) applies to the left panels in **E** and **F**. The scale bar (50 μm) applies to the right higher resolution magnified panels.

Figure S4. Experimental timeline for behavioral assessments.

(**A**) Overview of the experimental timeline for the behavioral tests, including the place-action test, object location memory (OLM), object recognition (ORM), open field and elevated plus maze (EPM) test. A one-week interval was maintained between the OLM, ORM and open field tests. Each experiment was conducted in a different behavioral room. CNO (5 mg/kg, i.p) or saline was administrated 30 min prior to the behavioral experiment each day. (**B**) Detailed timeline for the place-action test. Water deprivation was enforced throughout the duration of the experiments. Both test 1 and test 2 were repeated to ensure the reproducibility of the results. (**C**) Timeline for the OLM and ORM tests. CNO (5 mg/kg, i.p) or saline was administrated 30 min prior to the training session.

Figure S5. Reproducible impairment in place-action association from genetically targeted inactivation of M2-projecting RSCg neurons in a cross-maze decision-making task.

(**A**). Performance accuracy of M2 mice across two sets of Test 1 is depicted. Test 1 consisted of four days: saline on Day1, CNO on Day 3, saline on Day 5, and CNO on Day 7, with testing conducted every other day. All mice received CNO (5 mg/kg, i.p.) or saline 30 min before the first session on each testing day. The accuracy across total trials was calculated by dividing the total number of correct trials by a total of 56 trials from 8 sessions. Both sets of experiments show that hM4D/CNO mice exhibited significantly impaired accuracy compared to control mice.  Each light gray line and circle represents an individual mouse. Dark gray represents the mean percent accuracy of control mice with saline treatment, while pink represents AAV-hM4D-injected mice with CNO treatment. Data are presented as mean ± s.e.m., and the asterisks, *, and ** indicate significant differences as p < 0.05 and p< 0.01, respectively (Wilcoxon signed-rank test). **(B)** Mean percent accuracy of M2 group in two sets of Test 2 at novel locations is shown. Novel locations were A3. A4, B3 and B4 in Figure 6A. CNO inhibition mice exhibit a decrease in the accuracy of total trials in novel locations compared to saline control mice. (**C-D**) are arranged in the same way as the panels in **A-B**, to show the behavioral performance of the AD group in two sets of Tests 1 and 2. The results consistently show that CNO inhibition of AD-projecting RSCg neurons did not result in a significant reduction in performance accuracy in either familiar or novel locations, as compared to the control group. **Supplementary Table 1:** Viral information.

**Supplementary Table 2:** Quantitative analysis and statistical summary of viral tracing Experiments.

**Supplementary Table 3:** Quantitative analysis and statistical summary of behavioral experiments

**Supplementary Table 4**: Raw data for place-action tests.
