# Supplementary figures and images for: Projection-specific circuits of retrosplenial cortex with differential contributions to spatial cognition
Source: Mol Psychiatry. 2024 Nov 7;30(5):2068–84. doi: 10.1038/s41380-024-02819-8 (PMC12014379; doi:10.1038/s41380-024-02819-8)

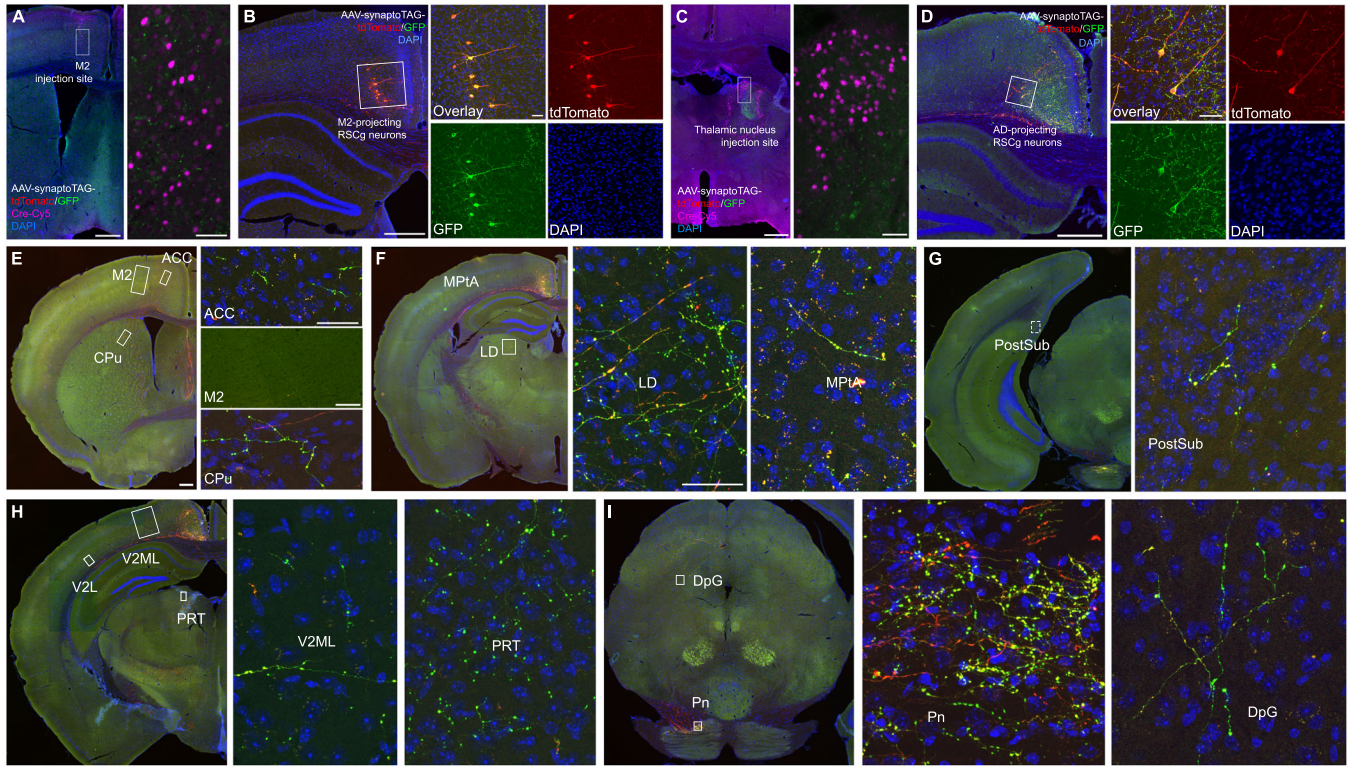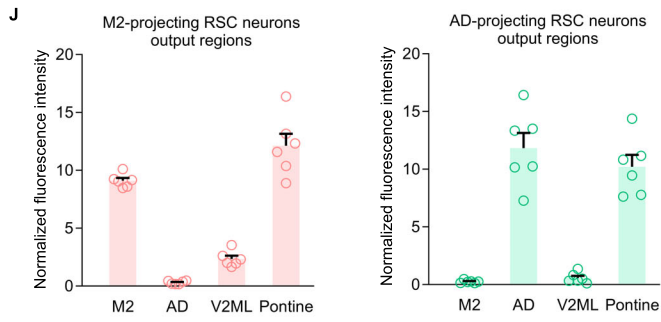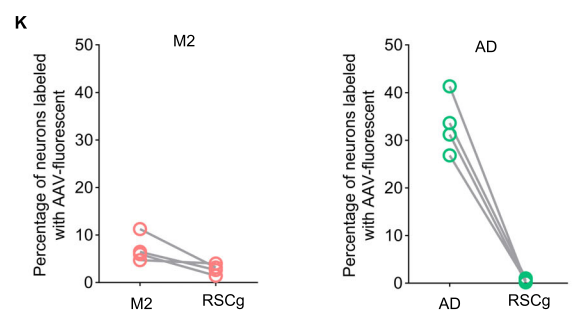

Supplement: Supplementary file 2 — Supplementary figure 1 [file 41380_2024_2819_MOESM2_ESM.pdf]

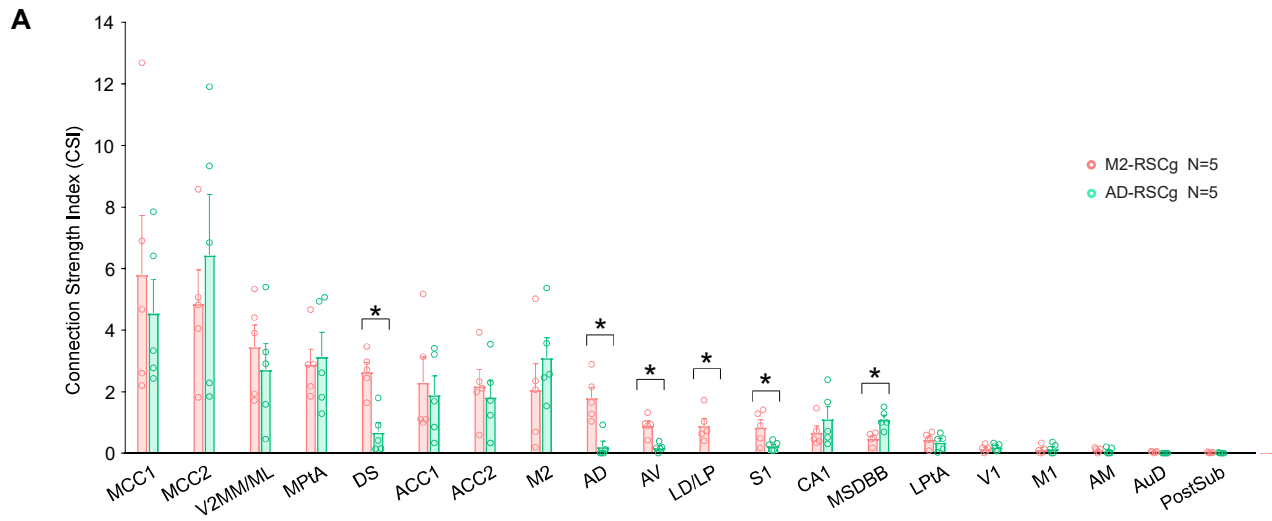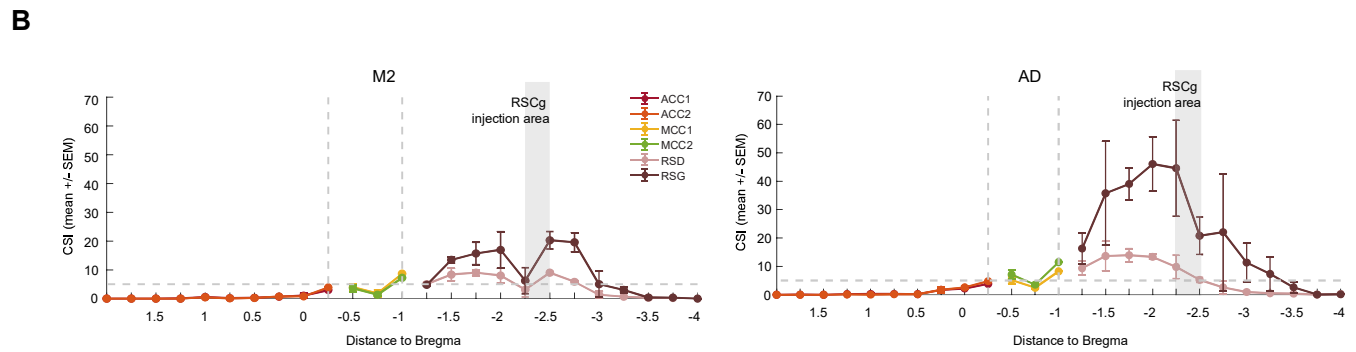

Supplement: Supplementary file 3 — Supplementary figure 2 [file 41380_2024_2819_MOESM3_ESM.pdf]

**A** Open field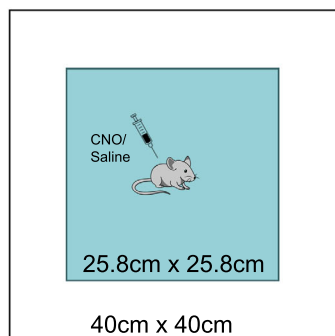**B**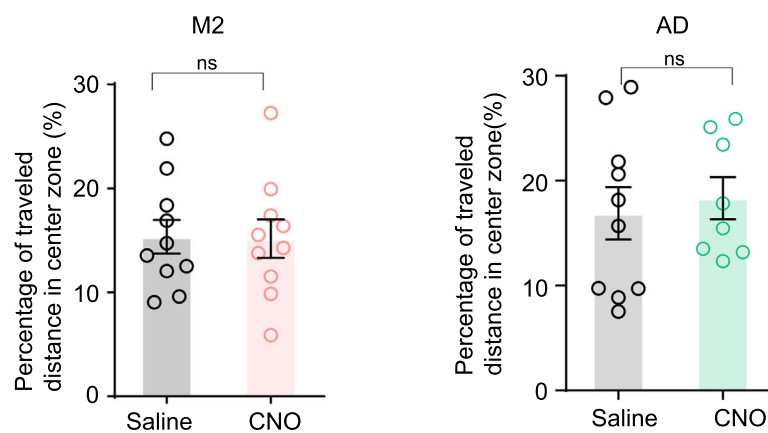**C** Elevated plus maze test (EPM)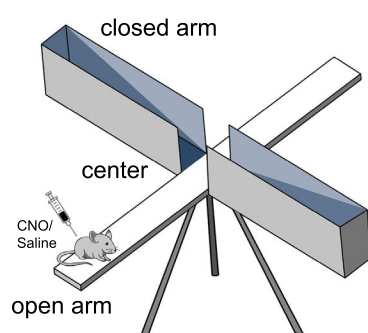**D**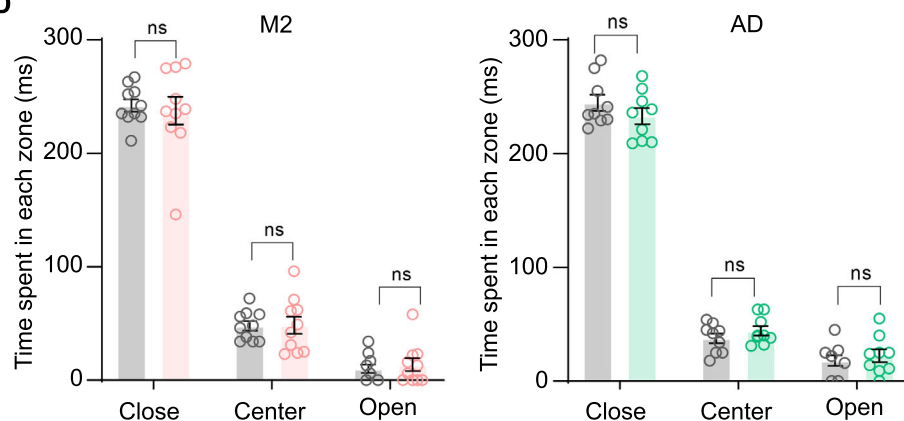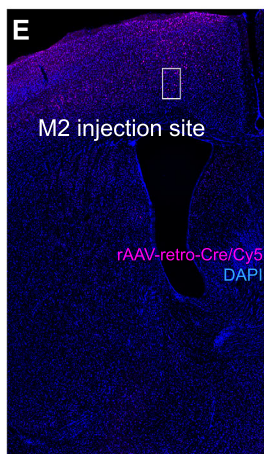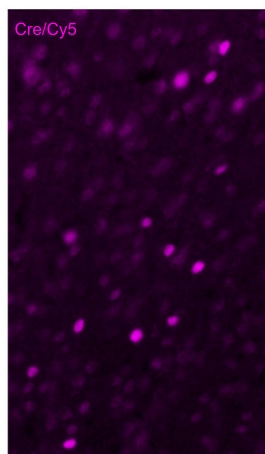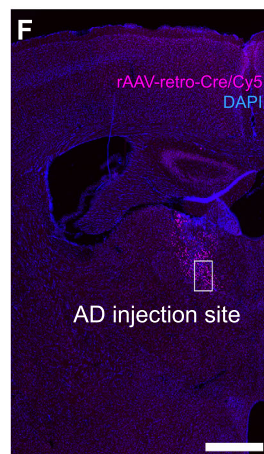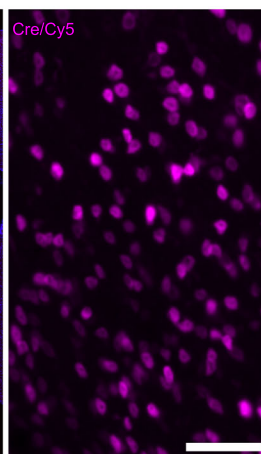

Supplement: Supplementary file 4 — Supplementary figure 3 [file 41380_2024_2819_MOESM4_ESM.pdf]

### M2-projecting RSCg inhibition

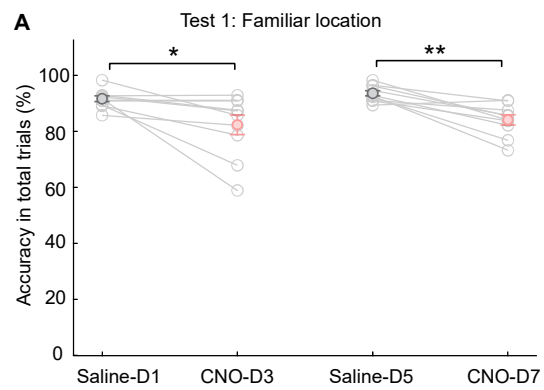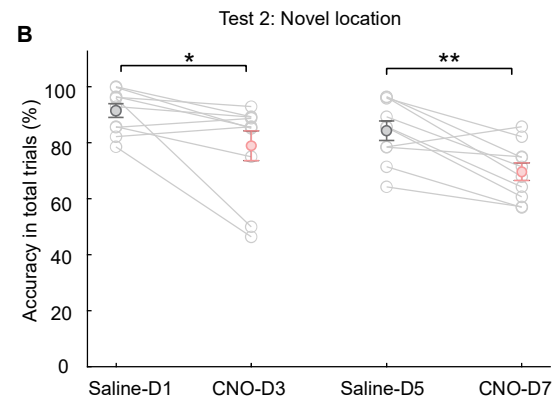

### AD-projecting RSCg inhibition

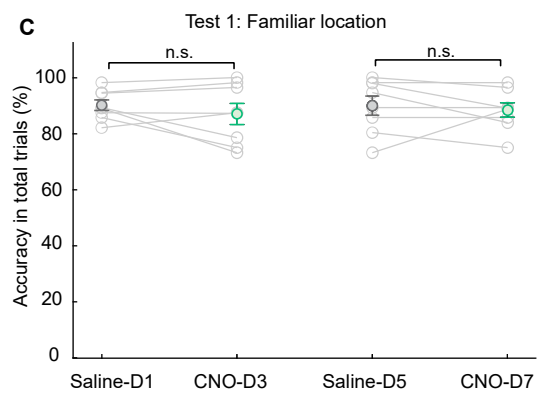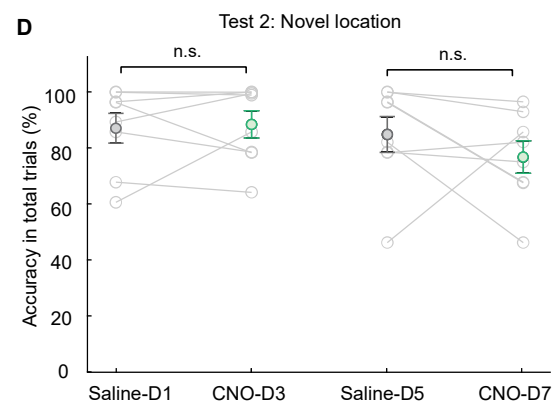

Supplement: Supplementary file 6 — Supplementary figure 5 [file 41380_2024_2819_MOESM6_ESM.pdf]
