# Supplementary material for: Projection-specific circuits of retrosplenial cortex with differential contributions to spatial cognition
Source: Mol Psychiatry. 2024 Nov 7;30(5):2068–84. doi: 10.1038/s41380-024-02819-8 (PMC12014379; doi:10.1038/s41380-024-02819-8)
Supplement: Supplementary file 5 — Supplementary figure 4 [file 41380_2024_2819_MOESM5_ESM.pdf]

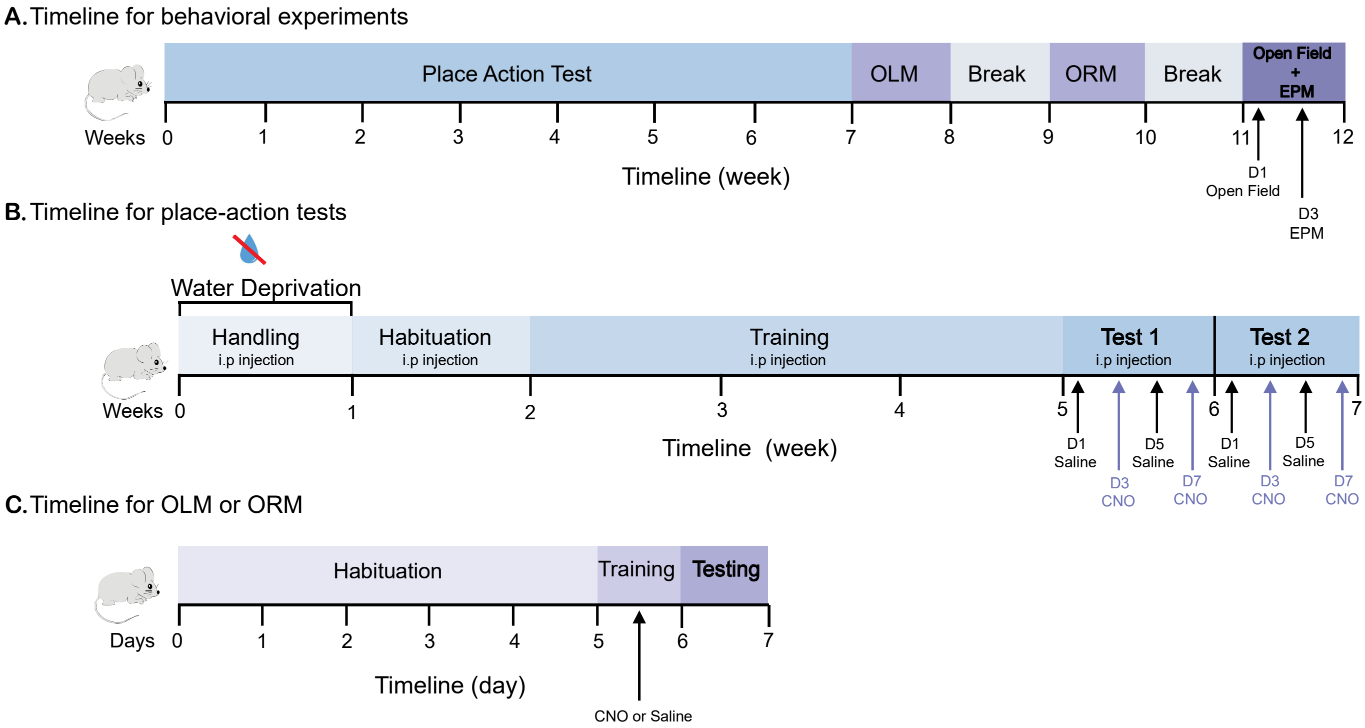
A. Timeline for behavioral experiments
Place Action Test
OLM
Break
ORM
Break
Open Field
+
EPM
Weeks
0
1
2
3
4
5
6
7
8
9
10
11
12
Timeline (week)
D1
Open Field
D3
EPM
B. Timeline for place-action tests
Water Deprivation
Handling
i.p injection
Habituation
i.p injection
Training
i.p injection
Test 1
i.p injection
Test 2
i.p injection
Weeks
0
1
2
3
4
5
6
7
Timeline (week)
D1
Saline
D3
CNO
D5
Saline
D7
CNO
D1
Saline
D3
CNO
D5
Saline
D7
CNO
C. Timeline for OLM or ORM
Habituation
Training
Testing
Days
0
1
2
3
4
5
6
7
Timeline (day)
CNO or Saline
